# AAV-mediated delivery of CRISPR/Cas9 targeting conserved overlapping ORFs efficiently suppresses HBV replication in hepatocyte models

**DOI:** 10.1016/j.btre.2026.e00961

**Published:** 2026-05-19

**Authors:** Pattida Kongsomboonchoke, Yongyut Pewkliang, Piyanoot Thongsri, Alisa Tubsuwan, Kanit Bhukhai, Nithi Asavapanumas, Phetcharat Phanthong, Suparerk Borwornpinyo, Wararat Chiangjong, Khanit Sa-ngiamsuntorn, Suradej Hongeng

**Affiliations:** a Department of Biotechnology, Faculty of Science, Mahidol University, Bangkok, 10400, Thailand; b Department of Pediatrics, Faculty of Medicine Ramathibodi Hospital, Mahidol University, Bangkok, 10400, Thailand; c Institute of Molecular Biosciences, Mahidol University, Nakhon Pathom, 73170, Thailand; d Department of Physiology, Faculty of Science, Mahidol University, Bangkok, 10400, Thailand; e Chakri Naruebodindra Medical Institute, Faculty of Medicine Ramathibodi Hospital, Mahidol University, Samutprakarn, 10540, Thailand; f Department of Anatomy, Faculty of Science, Mahidol University, Bangkok, 10400, Thailand; g Excellence Center for Drug Discovery, Faculty of Science, Mahidol University, Bangkok, 10400, Thailand; h Pediatric Translational Research Unit, Department of Pediatrics, Faculty of Medicine Ramathibodi Hospital, Mahidol University, Bangkok, 10400, Thailand; i Department of Biochemistry, Faculty of Pharmacy, Mahidol University, Bangkok, 10400, Thailand; j Biomarker and Epigenetic Science for Targeted Therapeutics (BEST) Research Center, Faculty of Pharmacy, Mahidol University, Bangkok, 10400, Thailand

**Keywords:** HBV, cccDNA, CRISPR/Cas9, AAV, Guide RNA, Hepatocytes, imHC

## Abstract

Chronic hepatitis B virus (HBV) infection remains a major global health burden due to the persistence of covalently closed circular DNA (cccDNA), which limits current antiviral therapies. We developed an adeno-associated virus (AAV)-delivered CRISPR/Cas9 system targeting conserved regions of the HBV genome. Three guide RNAs (gRNA1–3) targeting overlapping open reading frames of the surface antigen and polymerase genes were evaluated in HepG2.2.15 cells and HBV-infected hepatocyte-like cells (imHCs), with a reverse transcriptase-targeting gRNA and tenofovir alafenamide as controls. All gRNAs significantly reduced intracellular and extracellular HBV DNA levels and moderately decreased HBsAg secretion. Notably, gRNA2 induced a frameshift mutation and demonstrated superior antiviral efficacy, markedly reducing cccDNA levels, viral DNA levels, viral RNA levels, HBcAg expression, and HBsAg secretion with suppression maintained for up to 12 days. These findings highlight AAV-mediated CRISPR/Cas9 as a promising gene-based therapy for chronic HBV infection.

## Highlights

• Developed AAV-mediated CRISPR-Cas9 targeting conserved regions across HBV genotypes.
• Newly designed HBV-specific guide RNAs (gRNAs) with minimal off-target effects.
• gRNA2 induced frameshift mutations, significantly disrupting HBV gene function.
• gRNA2 outperformed RT gRNA and TAF, reducing HBV DNA, HBV RNA, cccDNA, and HBsAg.
• gRNA2 showed sustained antiviral effects, a promising chronic HBV therapy candidate.

## Graphical abstract

**Figure:**
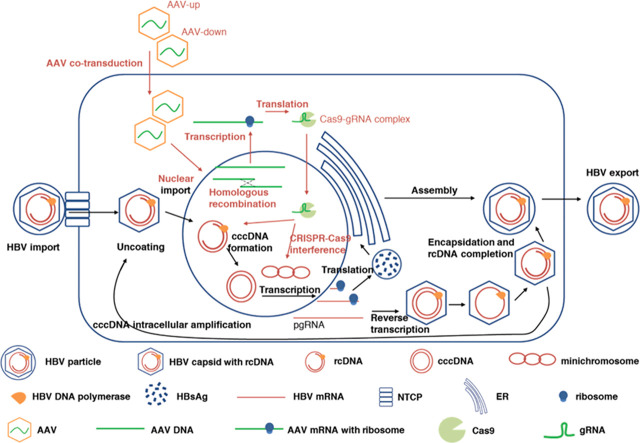

## 1 Introduction

Hepatitis B virus (HBV) infection is a major cause of inflammatory liver disease, affecting approximately 254 million chronically infected individuals worldwide, with an estimated 1.2 million new infections annually and 1.1 million deaths in 2022, mostly from cirrhosis and hepatocellular carcinoma (primary liver cancer) [1]. Chronic HBV carriers are at high risk of developing liver cirrhosis and hepatocellular carcinoma (HCC) [2,3], which are the leading causes of HBV-related mortality [1]. A key factor contributing to the persistence of chronic infection is the presence of covalently closed circular DNA (cccDNA), the transcriptional template of HBV within infected hepatocytes [4,5]. Because of its stable minichromosome-like structure [6,7] and its ability to self-amplify via intracellular recycling of mature core particles (a process known as intracellular amplification) [8], cccDNA can persist for decades. Currently, the two main classes of HBV therapeutics, pegylated interferon alpha (Peg-IFN) and nucleos(t)ide analog reverse transcriptase inhibitors (NUCs), do not effectively target cccDNA [9]. As a result, they fail to completely eliminate the virus in chronically infected individuals [[9], [10], [11]]. The structural resemblance of cccDNA to host chromosomal DNA, aside from nucleotide sequence differences, poses a significant challenge for its selective targeting. We employed an engineered nuclease system designed to selectively recognize and cleave HBV cccDNA to address this challenge.

One such promising tool is the clustered regularly interspaced short palindromic repeats (CRISPR) system and its associated endonuclease Cas9 (CRISPR/Cas9), originally derived from the bacterial adaptive immune system [[12], [13], [14]]. The CRISPR/Cas9 system has two essential components: a guide RNA (gRNA) and the Cas9 protein. The gRNA directs Cas9 to specific DNA sequences, enabling precise cleavage of double-stranded DNA. The induced DNA breaks are typically repaired by the non-homologous end joining (NHEJ) pathway, often resulting in insertions and deletions (indels) that disrupt gene function [15]. Due to its specificity and versatility, CRISPR/Cas9 is a powerful approach for targeting cccDNA and other replication-competent forms of HBV DNA in infected cells [16]. Among various delivery methods, adeno-associated virus (AAV) is one of the most effective and widely used vectors for delivery in gene therapy [17]. AAV is a non-pathogenic, replication-deficient virus capable of infecting both dividing and non-dividing cells and is considered safe for therapeutic use in gene therapy and genome editing [18,19]. However, its packaging capacity is limited to approximately 4.5–4.7 kb, while the Cas9 coding sequence from *Streptococcus pyogenes* is about 4.3 kb in size. To overcome this limitation, a dual-vector strategy [20] using two AAV vectors (designated AAV-up and AAV-down) was employed in this study.

Multiple strategies have been developed to design gRNAs targeting the HBV genome and cccDNA, with a common approach focusing on functional regions to ensure broad efficacy across diverse genotypes and strains. These regions encompass genes encoding the HBV X protein (HBx), DNA polymerase (Pol), core antigen (HBc), and surface antigen (HBs), as well as overlapping and regulatory elements, including the precore region, Enhancer I (EnhI), Enhancer II (EnhII), and the core promoter (CP) [[21], [22], [23], [24]]. The HBx open reading frame (ORF), essential for HBV RNA transcription, was also targeted in gRNA design [16,25]. Additionally, conserved motifs essential for viral replication, such as the YMDD motif in the HBV polymerase, have been effectively targeted to suppress HBV DNA synthesis [26]. Another approach utilizes computational tools to identify optimal gRNA sequences based on predicted efficiency and specificity. Commonly used platforms include the CRISPR Design Tool (Zhang Lab), the Broad Institute’s Genetic Perturbation Platform, CCTop, and BE-Designer [22,27,28]. A third strategy involves empirical screening of gRNAs based on their capacity to reduce HBV replication markers; such as hepatitis B surface antigen (HBsAg), hepatitis B e antigen (HBeAg), 3.5 kb HBV RNA, cccDNA, and S-RNA levels [21,27,29,30]. Although many of these gRNAs effectively suppress HBV replication, further optimization is required to achieve precise and efficient targeting of cccDNA.

In this study, we aimed to design gRNAs targeting conserved regions within early open reading frames (ORFs) of the HBV genome present in cccDNA. Targeting these regions is expected to induce frameshift mutations through CRISPR/Cas9-mediated double-strand breaks and NHEJ repair, thereby disrupting the function of entire genes. The three gRNA-targeting sites were selected from conserved genomic regions to ensure efficacy across pan-HBV genotypes, and computational predictions were used to identify the most effective candidates. To enhance delivery efficiency, CRISPR/Cas9 was administered using an AAV-based dual-vector system, employing two vectors designated AAV-up and AAV-down. In this study, HepG2.2.15 cells were used to evaluate the efficacy of gRNAs before assessing their anti-HBV activity in a natural infection model using HBVcc-infected imHC cells. Following CRISPR/Cas9 treatment, we evaluated the levels of cccDNA, intracellular and extracellular HBV DNA (viral load), HBsAg, and HBcAg to determine the extent of HBV replication disruption.

## 2 Materials and methods

### 2.1 Guide RNA design

Twelve complete hepatitis B virus (HBV) genome sequences, representing all known genotypes (A–J) and the *ayw* subtype, were retrieved from the NCBI nucleotide database (Table S1) and aligned using the biological sequence alignment editor (BioEdit) program to generate consensus sequences. Guide RNAs (gRNAs) were designed to be 20 nucleotides in length and positioned immediately upstream of the protospacer adjacent motif (PAM; 5′-NGG-3′), which is recognized by the commonly used *Streptococcus pyogenes* Cas9 nuclease. The web-based tool CRISPOR (version 5.01; http://crispor.tefor.net/, accessed May 8, 2022) was employed to assess gRNA specificity scores, predicted cleavage efficiency, and potential off-target sites in both the HBV and human genomes. Predicted off-target sites within the human genome were further validated using the verify guide design tool available on the SYNTHEGO website (https://design.synthego.com/#/validate, accessed February 1, 2024; Menlo Park, California), as well as the "Find Off-Targets by Seq" tool provided by the Wellcome Sanger Institute (https://www.sanger.ac.uk/, accessed February 1, 2024). All computational tools were used with default settings.

### 2.2 Plasmid construction and preparation

A 20-nucleotide gRNA (oligo 1) and its complementary strand (oligo 2) were synthesized with 5′-CCGG and 5′-AAAC overhangs, respectively. Equimolar amounts of each oligo (1 µL of 100 µM) were mixed with 8 µL of Guide-it Oligo Annealing Buffer (Takara Bio), resulting in a 10 µL reaction volume. The oligos were annealed by denaturation at 95°C for 2 min, followed by a gradual decrease in temperature from 85°C to 30°C for 10 min. The annealed product was then diluted 1:100 to a final concentration of 100 nM and stored at −20°C. For ligation, 15 ng of linearized pAAV-Guide-it-Down vector (Takara Bio) was mixed with 1 µL of the diluted annealed oligos, 5 µL of 2× DNA Ligation Mighty Mix (Takara Bio), and PCR-grade water to reach a final volume of 10 µL. The ligation reaction was incubated at 16°C overnight. The entire ligation mixture was then transformed into Stellar Competent Cells (Takara Bio) via heat shock transformation: incubation on ice for 30 min, heat shock at 42°C for 45 s, and immediate cooling on ice for 2 min. Transformed cells were recovered by adding 1 mL of SOC medium and shaking at 37°C for 1 h. Subsequently, cells were plated on LB agar containing 100 µg/mL ampicillin and incubated overnight at 37°C. Single colonies were selected, cultured in LB medium supplemented with 100 µg/mL ampicillin, and incubated overnight with vigorous shaking. According to the manufacturer's instructions, plasmids were purified using the NucleoBond Xtra Maxi Kit (cat. no. 740414.50; Macherey–Nagel). The purified plasmid DNA was diluted to a working concentration of 1 µg/µL.

### 2.3 AAVs production and extraction

HEK293T cells (CRL-3216™, ATCC) were seeded at 3 × 10⁶ cells per 100 mm dish and cultured to 80% confluency. AAVs were produced via co-transfection of three plasmids: either pAAV-Guide-it-Up or ligated pAAV-Guide-it-Down (encoding Cas9 fragments and gRNA), pRC2-mi342 (expressing Rep, Cap, and hsa-miR-342), and pHelper (expressing adenoviral E2A, E4, and VA genes). For each transfection, 13 µg of each plasmid was mixed in 600 µL Xfect Reaction Buffer (Takara Bio), followed by the addition of 11.7 µL Xfect Polymer. After 10 min of incubation, the transfection mix was added dropwise to HEK293T cells (Fig. S1). Following a 6-hour incubation at 37°C and 5% CO₂, the medium was replaced with DMEM supplemented with 2% FBS. The cells were further cultured for 3 days, harvested using 0.5 M EDTA (1/80 volume, pH 8.0), followed by incubating for 10 min at room temperature, and then pelleted by centrifugation. AAV particles were extracted using AAVpro extraction solution (Takara Bio), treated with DNase I (New England Biolabs) at 37°C for 15 min, and heat-inactivated at 75°C for 10 min. Viral genomes were released using an equal volume of lysis buffer (10 mM Tris-HCl, 25 mM EDTA, 1× SDS, 2% Triton X-100, 25 mM NaCl; pH 8.0) and incubated at 70°C for 10 min. AAV titers were quantified by qPCR using Cas9-specific primers (Cas9_F and Cas9_R; Table S2). Each 10 µL reaction included 5 µL of the KAPA SYBR FAST qPCR Kit (Kapa Biosystems), 0.2 µL of each primer, and 2 µL of diluted AAV template. Cycling was performed on a CFX96 Touch Real-Time PCR Detection System (Bio-Rad) with the following conditions: initial denaturation at 95°C for 3 min, followed by 40 cycles of 95°C for 3 s and 60°C for 30 s. Serial dilutions of pAAV-Guide-it-Up served as standards. AAV solutions were stored at −80°C until use.

### 2.4 Cell culture

HepG2.2.15 (SCC249, Sigma-Aldrich) and HEK293T (CRL-3216™, ATCC) cells were cultured in DMEM (Cytiva) supplemented with 10% fetal bovine serum (FBS; Cytiva), 100 U/mL penicillin, and 100 µg/mL streptomycin. The HepG2.2.15 medium contained 1% GlutaMAX™ and 380 µg/mL G418 (Thermo Fisher Scientific). Cells were maintained at 37°C in a humidified atmosphere with 5% CO₂, and the medium was changed every 2–3 days. All cell lines tested negative for mycoplasma contamination. The immortalized hepatocyte-like cell line (imHC) was established from human mesenchymal stem cells (hMSCs) genetically modified with hTERT and Bmi-1 via lentiviral vectors, as previously described [31]. Clones were selected based on high transgene expression, differentiated into hepatocyte-like cells using a three-step protocol, and validated by population doubling level and hepatic marker expression [32]. imHC cells were cultured in a 1:1 mixture of DMEM and Ham’s F12 (Gibco) with 10% FBS (HyClone), 1% GlutaMAX™ (Gibco), 100 U/mL penicillin, and 100 µg/mL streptomycin. Cells were maintained at 37°C, 5% CO₂, and subcultured every 3–5 days using 0.125% trypsin-EDTA.

### 2.5 Co-transduction of AAV-up and AAV-down in HepG2.2.15 and HBV-infected imHC cells

For HepG2.2.15 cells, 3 × 10⁵ cells were seeded per well in 6-well plates and incubated for 18 h. AAV-up and AAV-down vectors were thawed, mixed at a 1:1 genome ratio, and added at a multiplicity of infection (MOI) of 2 × 10⁶ in a volume less than one-tenth of the culture medium. The AAV mixture was added dropwise, and the plates were gently rocked to ensure even distribution. Cells were incubated with AAV for 24 h, and the effects of CRISPR/Cas9 treatment were evaluated on days 3, 6, 9, and 12 post-transduction. To establish the infection model, one million imHC cells were seeded per well in 6-well plates for HBV infection and cultured overnight. Before infection, imHCs were maturated by culturing in differentiation medium containing 2% DMSO (Sigma) for two weeks [33]. Differentiated imHCs (d-imHCs) were washed with DPBS and incubated with 1 mL of serum-free Williams’ E medium containing 4% PEG 8000 (Sigma) and antibiotics. For HBVcc (cell culture strain) infection, 10 µL of HBV viral stock (10⁸ genome equivalents/mL) was added at a multiplicity of infection (MOI) of 1 and incubated for 24 h at 37°C. Post-infection, cells were washed thoroughly with DPBS and maintained in Williams’ E complete medium supplemented with 10% FBS, 100 U/mL penicillin, 100 µg/mL streptomycin, 2 mM GlutaMAX™ (Gibco). Three days after infection, AAV-up and AAV-down vectors were thawed at 37°C, mixed at a 1:1 ratio based on genome titer, and added to the HBV-infected d-imHCs at a total volume equal to one-tenth of the culture medium. Transduction was carried out for 24 h, followed by replacement with fresh complete medium, which was refreshed every three days until day 12 post-transduction. Non-infected cells served as negative controls, and non-transduced infected cells were positive controls. Tenofovir alafenamide (TAF) was added on day 4 post-infection as a treatment control. The antiviral effect of CRISPR/Cas9 was assessed by measuring intracellular HBV DNA, viral load, cccDNA, HBsAg, and HBcAg levels.

### 2.6 Quantification of intracellular HBV DNA and viral load by real-time qPCR

HBV DNA levels were determined as previously described [33,34]. In brief, total intracellular DNA was isolated from HepG2.2.15 cells or HBV-infected imHC using the NucleoSpin Tissue Kit (Macherey-Nagel), while extracellular HBV DNA was extracted from conditioned culture media using the NucleoSpin Blood Kit (Macherey-Nagel), according to the manufacturer’s instructions. Quantitative real-time PCR (qPCR) was performed using 100 ng of total intracellular DNA or 2 µL of extracted extracellular HBV DNA as template, employing the KAPA SYBR FAST qPCR Kit (KAPA Biosystems) on a CFX96 Touch Real-Time PCR Detection System (Bio-Rad). HBV-specific primers (Table S2) were used to amplify viral DNA. Absolute quantification was achieved using a standard curve generated from serial dilutions of the HBV 1.3-mer wild-type replicon plasmid (Addgene plasmid #65459). PRNP was used as an internal reference gene (Table S2). PCR cycling conditions consisted of an initial denaturation at 95°C for 3 min, followed by 40 amplification cycles of denaturation at 95°C for 3 s and annealing/extension at 60°C for 20 s. Each sample was analyzed in triplicate. Amplicons were confirmed by gel electrophoresis and melting curve analysis. The fold change of intracellular HBV DNA was calculated using the 2^–ΔΔCt^ method, normalized to PRNP.

### 2.7 Quantification of HBV RNA by real-time PCR

HBV RNA levels were measured using a protocol adapted from previously described method [35]. Briefly, total RNA was extracted from cell pellets using the *illustra* RNAspin Mini Kit (GE Healthcare) and reverse transcribed with the ImProm-II™ System (Promega) using random primers. The resulting cDNA was diluted 1:6 for qPCR. HBV total RNA, pgRNA, and preS1 RNA were quantified using specific primers (Table S2) and the KAPA SYBR® FAST qPCR Kit (Kapa Biosystems). Reactions contained 2 µL of diluted cDNA and 0.2 µM primers, and were run on a CFX96 Touch System (Bio-Rad) with the following conditions: 95°C for 3 min, followed by 40 cycles of 95°C for 3 s and 60°C for 20 s. Each sample was analyzed in triplicate. Amplicons were confirmed by gel electrophoresis and melting curve analysis. The relative mRNA expression was calculated using the 2^–ΔΔCt^ method, normalized to GAPDH**.**

### 2.8 Detection of HBV cccDNA by exonuclease treatment and PCR

To detect cccDNA in HBV-infected imHCs following AAV-mediated CRISPR/Cas9 transduction, relaxed circular DNA (rcDNA) was selectively removed from the extracted HBV DNA using the NucleoSpin Plasmid DNA Extraction Kit (MN, Düren, Germany) [33,34]. To selectively enrich for covalently closed circular DNA (cccDNA), extracted DNA was treated with exonuclease V to digest non-cccDNA viral DNA species, as previously described [33,34]. cccDNA was amplified using specific primers that generate a 580-bp PCR product spanning the gap and nick regions of the relaxed circular (rc) HBV genome, enabling selective detection of cccDNA [36,37]. Primers for HBV cccDNA are listed in Table S2. Each 10 µL qPCR reaction contained 5 µL of 2× KAPA SYBR® FAST qPCR (Kapa Biosystems), 0.2 µL of each primer (10 µM), 2 µL of DNA template, and nuclease-free water to a final volume of 10 µL. Quantitative PCR was performed using a CFX96 Touch Real-Time PCR Detection System (Bio-Rad) under the following cycling conditions: initial denaturation at 95°C for 3 min, and 40 cycles of 95°C for 3 s and 60°C for 35 s. Quantification of cccDNA levels was performed using a standard curve generated from serial dilutions of the HBV 1.3-mer wild-type replicon plasmid (Addgene plasmid #65459).

### 2.9 Quantification of HBV cccDNA by droplet digital PCR (ddPCR)

Detection and quantification of HBV cccDNA were previously validated using droplet digital polymerase chain reaction (ddPCR) [38]. Total DNA was extracted from infected imHC cells using the NucleoSpin Tissue Kit (Macherey–Nagel). One microgram of DNA was subsequently digested with EcoRI (Promega), which specifically cleaves the HBV subtype *ayw* genome while preserving the reference amplicon region. Quantification of cccDNA by ddPCR was performed as previously described [39]. Briefly, each 25 µL ddPCR reaction comprised 12.5 µL of 2× ddPCR Supermix for Probes (Bio-Rad), 900 nM of each forward and reverse primer, 250 nM hydrolysis probes for HBV cccDNA and the reference gene RPP30 (sequences listed in Table S2), and 1 µL of digested DNA. For droplet generation, 20 µL of the reaction mixture and 70 µL of Droplet Generation Oil were loaded into DG8™ cartridges (Bio-Rad), sealed with DG8™ gaskets, and processed using a QX200™ Droplet Generator (Bio-Rad). Approximately 40 µL of droplets were then transferred to a 96-well PCR plate and heat-sealed with pierceable foil at 180°C for 5 s. Thermal cycling was performed on a T100™ Thermal Cycler (Bio-Rad) under the following conditions: initial denaturation at 95°C for 10 min; 45 cycles of 95°C for 30 s and 58°C for 1 min; followed by enzyme deactivation at 98°C for 10 min. After amplification, droplets were analyzed using a QX200™ Droplet Reader, and data were processed with QuantaSoft™ software (Bio-Rad). HBV cccDNA copy number variation (CNV) was calculated as: CNV = 2 × (cccDNA concentration / RPP30 concentration).

### 2.10 Indirect immunofluorescence assay

At day 7 post-transduction, cells were fixed with 4% paraformaldehyde and permeabilized in blocking buffer containing 3% bovine serum albumin (BSA) and 0.2% Triton X-100 in PBS for 1 h at room temperature. Cells were incubated overnight at 4°C with an anti-HBV core antigen (HBcAg) primary antibody (1:100; ab8637, Abcam), followed by incubation with Alexa Fluor® 568-conjugated goat anti-mouse IgG secondary antibody (1:500; A-11004, Invitrogen) for 1 h at room temperature. Nuclei were counterstained with Hoechst 33342 (1:1000; Thermo Fisher Scientific). Fluorescence images were acquired using an Olympus IX81 inverted fluorescence microscope at 20× magnification.

### 2.11 HBsAg detection using an enzyme-linked immunosorbent assay (ELISA)

Secreted hepatitis B surface antigen (HBsAg) in culture supernatants was quantified using a commercial HBsAg ELISA kit (Cat. No. KA0286; Abnova, Taipei, Taiwan), according to the manufacturer’s instructions. Negative controls, positive controls, and blanks were included in the assay, with three wells allocated for negative controls, two wells for positive controls, and one well for the blank. Absorbance was measured 10 min after the addition of the stop solution using an EnVision Multilabel Reader (PerkinElmer, Shelton, USA) at 450 nm, with 620 nm used as the reference wavelength.

### 2.12 On-target DNA sequencing

Primer pairs were designed to flank each CRISPR/Cas9 target site for on-target amplicon sequencing. Genomic DNA templates were prepared from HepG2.2.15 cells as described above. Target regions were amplified by conventional PCR using a high-fidelity DNA polymerase. Each 25 µL PCR reaction contained 12.5 µL Q5 Hot Start High-Fidelity 2× Master Mix (New England Biolabs, Ipswich, MA, USA), 1.25 µL of each primer (10 µM; HBV_Fragment_1883_F and HBV_Fragment_1883_R), 2.5 µL of genomic DNA template, and nuclease-free water to a final volume of 25 µL. The sequences of the HBV_Fragment_1883_F and HBV_Fragment_1883_R primers are provided in Table S2. PCR amplification was performed under the following cycling conditions: initial denaturation at 98°C for 30 s; 40 cycles of denaturation at 98°C for 5 s, annealing at 60°C for 30 s, and extension at 72°C for 15 s; followed by a final extension at 72°C for 5 min. PCR products were resolved by agarose gel electrophoresis, and bands of the expected size were excised and purified using the NucleoSpin Gel and PCR Clean-up Kit (Cat. No. 740609.50; Macherey-Nagel), according to the manufacturer’s instructions. Purified amplicons were subjected to Sanger sequencing (U2Bio, Seoul, Republic of Korea). Sequence chromatograms were analyzed using BioEdit software. Contig assembly was performed using the CAP contig assembly program with default parameters, and multiple sequence alignments of on-target amplicons were generated using ClustalW with default settings.

### 2.13 Peptide structure prediction of target genes encoding proteins

Edited nucleotide sequences of on-target genes were translated *in silico* into amino acid sequences using the Expasy Translate tool (https://web.expasy.org/translate/) [40]. The resulting amino acid sequences were used for 3D structural modeling via SWISS-MODEL (https://swissmodel.expasy.org/interactive), and binding functions were predicted using PredictProtein (https://predictprotein.org/) [41] to assess potential binding activity. All tools were used with default parameters.

### 2.14 T7 endonuclease I (T7E1) assay

Primer pairs were designed to flank the CRISPR/Cas9 target sites for on-target amplicon generation. Genomic DNA templates were prepared from HBV-infected imHCs transduced with AAV-mediated CRISPR/Cas9, as described above. On-target DNA fragments (717 bp) were amplified by conventional PCR using a high-fidelity DNA polymerase. Each 25 µL PCR reaction contained 12.5 µL Q5 Hot Start High-Fidelity 2× Master Mix (New England Biolabs, Ipswich, MA, USA), 1.25 µL of each primer (10 µM; HBV_Fragment_717_F and HBV_Fragment_717_R), 2.5 µL of genomic DNA template, and nuclease-free water to a final volume of 25 µL. The sequences of the HBV_Fragment_717_F and HBV_Fragment_717_R primers are provided in Table S2. PCR amplification was performed under the following cycling conditions: initial denaturation at 98°C for 30 s; 40 cycles of denaturation at 98°C for 5 s, annealing at 60°C for 30 s, and extension at 72°C for 15 s; followed by a final extension at 72°C for 5 min. PCR products were confirmed by agarose gel electrophoresis, and amplicons of the expected size were purified using the NucleoSpin Gel and PCR Clean-up Kit (Cat. No. 740609.50; Macherey–Nagel), according to the manufacturer’s instructions. Purified PCR products (250 ng) were denatured at 95°C for 5 min and gradually reannealed to form heteroduplex DNA. The reannealed products were digested with 10 U of T7 endonuclease I (M0302; New England Biolabs) at 37°C for 20 min and subsequently analyzed on a 1% agarose gel. Cleavage band intensities were quantified using ImageJ software (version 1.46).

### 2.15 Statistical analysis

Experiments were performed in triplicate. Data were presented as mean ± standard deviation. Statistical analysis was performed using GraphPad Prism 9 software (GraphPad Software Inc., Boston, USA). Experimental data were analyzed by one-way ANOVA with Dunnett’s test for comparisons against a single control group, at *p*-value < 0.05 considered statistically significant.

## 3 Results

### 3.1 Design of gRNAs targeting HBV consensus sequences in early open reading frames

**Fig. 1:**
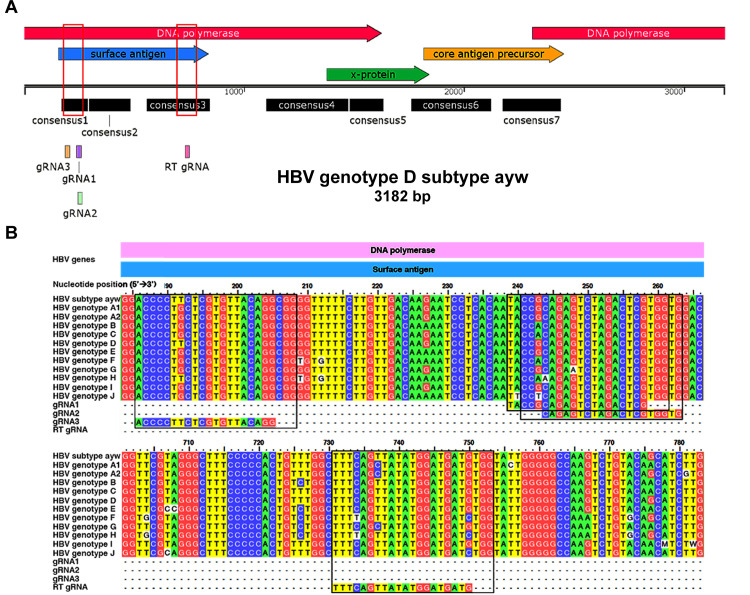
**Design of gRNAs targeting the early ORF of HBsAg, overlapping the middle ORF of the HBV DNA polymerase.** (**A**) Genomic positions of gRNAs on the HBV genotype D, subtype *ayw*. The gRNAs were designed within consensus regions of overlapping genes. A previously reported RT gRNA [26] was included as a positive control. Color boxes on the HBV map indicate the regions (matching colors) shown at higher magnification in panel (**B**). (**B**) Multiple sequence alignment of gRNA target sites with HBV genomes from genotypes A–J, showing that the gRNAs target conserved regions across all genotypes. The four black boxes highlight the alignment of each gRNA and its respective PAM sequence.

Guide RNAs (gRNAs) were primarily designed based on the complete genome of HBV genotype D, subtype *ayw* (GenBank accession no. U95551.1), which is the same strain present in HepG2.2.15 cells [42]. The HBV genome contains four open reading frames (ORFs), several of which overlap (Fig. 1A). To identify conserved target sites, whole-genome sequences from HBV genotypes A–J and subtype *ayw* were aligned using ClustalW in BioEdit. This analysis revealed seven conserved regions across the genome: HBsAg/Pol overlap, Pol, X/Pol overlap, late X ORF, Core/X overlap, Core, and Core/Pol overlap. These overlapping regions were of particular interest, as CRISPR/Cas9-mediated editing could disrupt multiple genes simultaneously. Targeting the X/Pol overlap region (consensus 4 and 5) might, for example, affect the X gene while sparing Pol, as the location is toward the 3′ end of the Pol ORF. Conversely, the Core/Pol overlap (consensus 7) is more likely to affect Pol than Core. Notably, the HBsAg/Pol overlap, particularly consensus region 1, was selected for its ability to target the early ORF of HBsAg and the central region of the Pol gene. Three gRNAs (gRNA1, gRNA2, and gRNA3) were designed to target these conserved sequences (Fig. 1A). All three were identical to the HBV genotype D subtype *ayw* reference sequence and showed high sequence similarity to other genotypes (Fig. 1B). Each gRNA contained a highly conserved seed region located 5–10 nucleotides upstream of the PAM sequence. As controls, a positive control gRNA targeting the HBV reverse transcriptase active site (RT gRNA) was used, which has been shown to completely suppress HBV DNA release [26], while a negative control gRNA targeting the human Ankyrin repeat domain (ANKRD gRNA), which does not target the HBV genome, was included. To minimize off-target effects, all gRNAs were designed for high specificity to HBV sequences with minimal homology to the human genome. CRISPOR analysis confirmed that all gRNAs had no predicted off-targets within the HBV genome and exhibited on-target cleavage efficiency scores >50 (Table 1). Off-target predictions in the human genome were further validated using two independent tools. First, SYNTHEGO identified potential on- and off-targets only for the ANKRD gRNA (Fig. S2, Table S3A). Second, the "Find Off-targets by Seq" tool confirmed that no HBV-specific gRNAs had complete matches within the human genome. Notably, gRNA1 exhibited four mismatches at potential off-target sites and had the lowest predicted off-target risk among the three HBV-targeting gRNAs (Table S3B). Although mismatches between gRNAs and human DNA generally reduce Cas9 cleavage activity [43], off-target effects in functionally critical genomic regions could still pose safety concerns. Therefore, comprehensive off-target validation is essential before any clinical application.

**Table 1:** Predicted scores and off-target in HBV and the human genome of the designed gRNAs.

**gRNAs**	**ORFs**	**Strand**	**Guide sequence** ***PAM***	**Specificity score**^a^	**Predicted efficiency**^b^	**Off-targets in HBV genotype D, AB554016.1**	**Off-targets in the human genome (hg19)**
gRNA1	Pol overlap Sur	-	TACCGCAGAGTCTAGACTCG *tgg*	100	66	1-0-0-0-0	0-0-0-0-26
gRNA2	Pol overlap Sur	+	CACCACGAGTCTAGACTCTG *CGG*	100	72	1-0-0-0-0	0-0-0-3-87
gRNA3	Pol overlap Sur	-	ACCCCTTCTCGTGTTACAGG *cgg*	100	57	1-0-0-0-0	0-0-0-2-43
RT gRNA	Pol overlap Sur	-	TTTCAGTTATATGGATGATG *tgg*	100	62	1-0-0-0-0	0-0-1-13-209
ANKRD gRNA	No target found		TCTGACAAGGGCTGTAAGTG *NGG*	Not specified	Not specified	0-0-0-0-0	1-0-0-13-246

The last 2 columns are off-targets for 0-1-2-3-4 mismatches. For each number of mismatches, the number of off-targets is indicated.

a The specificity score measures the uniqueness of a guide in the genome, ranging from 0-100, with 100 being the best [60]. No cut-off value was determined.

b The predicted efficiency provides how well the target is cleaved, ranging from 0-100, with 100 being the best [61]. No cut-off value was determined.

### 3.2 Inhibition of HBV replication in HepG2.2.15 cells by AAV-delivered CRISPR-Cas9 system

**Fig. 2:**
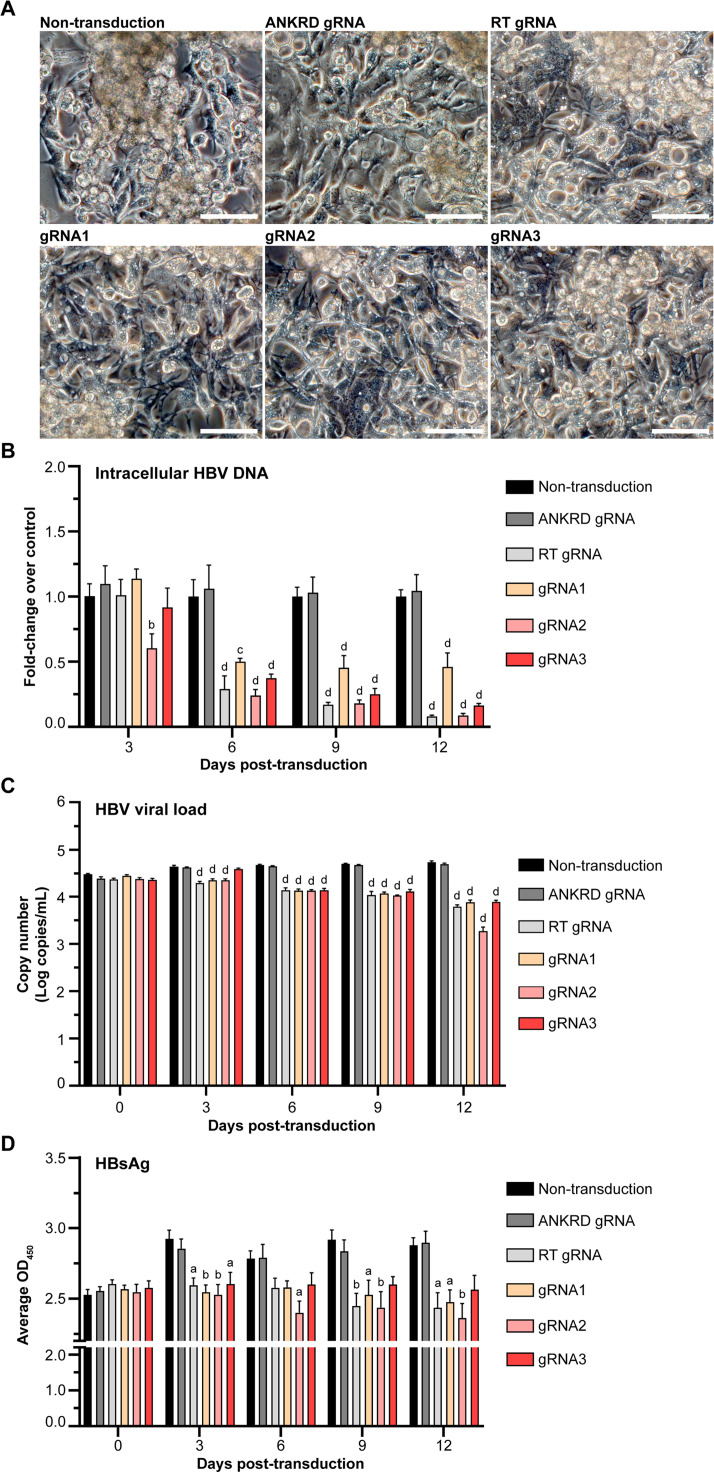
**Effect of AAV-delivered CRISPR/Cas9 with individual gRNAs on HBV replication in HepG2.2.15 cells.** (**A**) Morphology of HepG2.2.15 cells at day 12 post-transduction. Images were captured at 20× magnification; scale bars represent 50 µm. (**B**) Relative quantification of intracellular HBV DNA by qPCR normalized to PRNP was expressed as fold change compared to non-transduced cells of each post-transduction time point. (**C**) Absolute quantification of extracellular HBV DNA (viral load) by qPCR, using a standard curve generated from a known concentration of HBV 1.3-mer WT replicon plasmid. (**D**) Quantification of HBsAg by ELISA. Three biological replicates were analyzed. Statistical significance was determined, and the letters (a, b, c, and d) represent statistical differences between the treatments and their respective controls with a *p*-value less than 0.05, 0.01, 0.001, and 0.0001, respectively.

To evaluate the antiviral effects of the CRISPR/Cas9 system on HBV replication, intracellular and extracellular HBV DNA levels as well as secreted HBsAg were assessed every three days up to day 12 post-transduction. AAV-mediated delivery of CRISPR/Cas9 encoding any of the three gRNAs did not alter the morphology of HepG2.2.15 cells. Transduced cells maintained typical morphology and formed multilayered adherent cultures by day 12, comparable to non-transduced controls (Fig. 2A). Morphologically, adherent cells exhibited fibroblast-like features with bi- or multipolar elongated shapes, whereas the upper cell layer consisted of clustered, lymphoblast-like cells. HBV replication was first evaluated by quantifying intracellular HBV DNA, normalized to PRNP and expressed as fold changes relative to non-transduced cells at each time point (Fig. 2B). Cells transduced with CRISPR/Cas9 carrying gRNA2 exhibited an early reduction in intracellular HBV DNA to approximately 0.6-fold as early as day 3 post-transduction, whereas other groups showed no significant changes at this time point. From day 6 to day 12 post-transduction, cells transduced with CRISPR/Cas9 carrying the RT gRNA or any of the three designed gRNAs demonstrated significant reductions in HBV DNA levels compared with non-transduced cells. Among the designed gRNAs, gRNA2 showed the most pronounced suppression of intracellular HBV DNA, comparable to that observed with the RT gRNA (positive control). In contrast, cells transduced with the ANKRD gRNA showed no significant change in HBV DNA levels throughout the experimental period. To assess viral particle release, extracellular HBV DNA levels were quantified in culture supernatants at three-day intervals up to day 12 post-transduction (Fig. 2C). Prior to transduction (day 0), all groups exhibited similar viral loads of approximately 4.3 log copies/mL. In non-transduced cells and ANKRD gRNA-transduced cells, extracellular HBV DNA levels increased progressively over time, indicating active HBV replication in HepG2.2.15 cells. By day 3 post-transduction, cells transduced with CRISPR/Cas9 carrying the RT gRNA or gRNA1 and gRNA2, but not gRNA3, exhibited a reduction in extracellular HBV DNA levels. From day 6 to day 12 post-transduction, all CRISPR/Cas9-transduced groups carrying the RT gRNA or the designed gRNAs showed significant inhibition of HBV release. Notably, cells transduced with CRISPR/Cas9 carrying gRNA2 exhibited a marked reduction in extracellular HBV DNA from 4.7 to 3.2 log copies/mL by day 12 post-transduction (Fig. 2C). Secreted HBsAg levels were measured in culture supernatants by ELISA (Fig. 2D). HBsAg production was significantly reduced in cells transduced with CRISPR/Cas9 carrying the RT gRNA or any of the three designed gRNAs from early post-transduction onward compared with non-transduced control. Among these, CRISPR/Cas9 carrying gRNA2 resulted in sustained and pronounced suppression of HBsAg secretion throughout the experimental period (Fig. 2D). Collectively, these results demonstrate that AAV-delivered CRISPR/Cas9 constructs encoding the RT gRNA or the newly designed HBV-targeting gRNAs effectively suppress HBV replication in HepG2.2.15 cells by reducing both intracellular and extracellular HBV DNA levels and inhibiting HBsAg production. The sustained antiviral activity observed with gRNA2 highlights its potential as a promising candidate for further evaluation in HBV infection models.

### 3.3 Structural protein prediction following HBV DNA nucleotide deletion

**Fig. 3:**
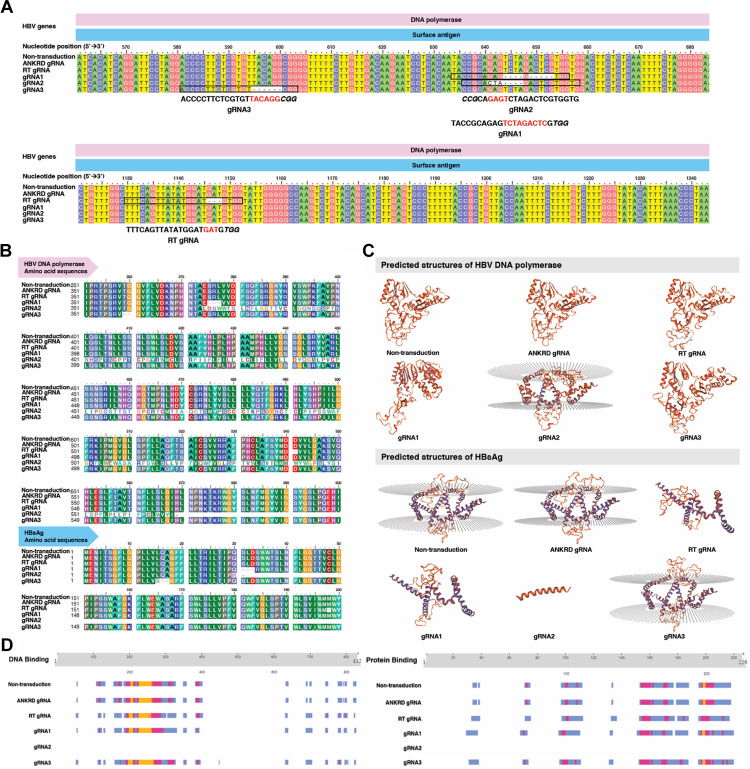
**HBV DNA sequencing and predicted effects on amino acid sequences, protein structures, and functions.** (**A**) DNA sequencing results showing CRISPR/Cas9-induced deletions. gRNA sequences indicate the binding position of HBV DNA. Red alphabets on gRNA sequences show gap regions and the PAM sequence, respectively. (**B**) Predicted amino acid sequences from the edited HBV DNA polymerase and HBsAg reading frames. Only mutated regions are shown; dashes (–) represent gaps. (**C**) Predicted 3D protein structures of HBV DNA polymerase and HBsAg, and (**D**) functional annotations of binding capabilities, particularly DNA and protein binding, of HBV DNA polymerase and HBsAg after CRISPR/Cas9 editing. Color bars indicate the reliability index (RI): blue (0–33), red (34–66), and yellow (67–100).

Following transduction of HepG2.2.15 cells with AAV vectors carrying CRISPR/Cas9 and specific gRNAs, mutations in HBV genomic DNA were assessed via on-target sequencing at 12 days post-transduction. Full-length amplicons were assembled using the Contig Assembly Program (CAP) and aligned using ClustalW. All CRISPR/Cas9 systems, including those with designed gRNAs and RT gRNA, induced deletions at the predicted sites (Fig. 3A), ranging from 3 to 9 nucleotides near the PAM sequence. For instance, CRISPR/Cas9 with gRNA1 caused a 9-nucleotide deletion, either as a continuous sequence (TCTAGACTC) or discontinuous segments (TCTA and ACTCG). Similarly, deletions of 3, 4, or 6 nucleotides were observed with RT gRNA, gRNA2, and gRNA3, respectively. To assess the functional impact, edited DNA sequences were translated using the Expasy Translate tool [40] and aligned in BioEdit, using the reading frames of HBsAg or HBV polymerase as appropriate. The 9-nucleotide deletion from gRNA1 led to the loss of three amino acids in both HBsAg (Serine, Leucine, and Aspartic acid) and HBV DNA polymerase (Serine, Arginine, and Leucine) (Fig. 3B). Additionally, serine was substituted by arginine in the HBsAg sequence. Similar two-amino acid deletions were predicted for gRNA3 in both proteins without inducing a frameshift. In contrast, gRNA2 introduced a frameshift and a premature stop codon, substantially truncating the translated protein. Homology modeling of predicted amino acid sequences was performed using SWISS-MODEL [41] (Fig. 3C). The HBV polymerase structures from non-transduced and gRNA1/RT gRNA/gRNA3-edited cells aligned with the reverse transcriptase/ribonuclease H domain (SMTL ID: 4ol8.1), consistent with HBV genotype D, subtype *ayw*. Although the HBsAg protein structures were generally conserved, conformational differences were noted. Notably, gRNA2-edited sequences showed significant structural alterations. The predicted HBsAg protein (SMTL ID: 7tuk.1.A) differed from the original structure, with CRISPR/Cas9-gRNA2 editing resulting in only a short helical form due to the premature stop codon (Fig. 3C).

PredictProtein (https://predictprotein.org/) was applied to validate these structural predictions. For non-transduced HBV polymerase, the closest match was protein P (S6A6W5) with 0.86 identity across 845 residues (Table 2). For HBsAg, Q4KZQ5 (identity 0.94, 226 residues) was identified (Table 2). All edited sequences matched their respective proteins except for the HBV polymerase from gRNA2-edited cells, which was predicted to be a large envelope protein, indicating potential loss of function. Protein and DNA-binding activities were further evaluated using the ProNA2020 module of PredictProtein [44]. Binding functions in CRISPR/Cas9-edited cells (RT gRNA, gRNA1, and gRNA3) were comparable to those of non-edited controls and ANKRD gRNA-transduced cells (Fig. 3D). However, no binding activity was predicted for the gRNA2-edited HBsAg or HBV polymerase, suggesting a complete loss of function due to structural disruption.

**Table 2:** HBV proteins are predicted from amino acid sequences.

**Protein name**	**CRISPR/Cas9 system containing gRNAs**	**UniProt ID prediction** (reference sequences)	**Predicted protein**	**Identity**^a^	**Expected value**^b^	**Matched length**^c^
HBV DNA polymerase	Non-transduction	S6A6W5	Protein P	0.86	0.00	845
	ANKRD gRNA			0.86	0.00	845
	RT gRNA	Q80IL1		0.86	0.00	842
	gRNA1			0.86	0.00	842
	gRNA2	G1E7D5	Large envelope protein	0.80	0.00	569
	gRNA3	Q80IL1	Protein P	0.86	0.00	842
HBsAg	Non-transduction	Q4KZQ5	HBsAg protein	0.94	< 10e-50	226
	ANKRD gRNA			0.94	< 10e-50	226
	RT gRNA			0.93	< 10e-50	226
	gRNA1	A0A1Y0DIG3	Large envelope protein	1.00	< 10e-50	147
	gRNA2	D0EEY6		0.86	2e-04	29
	gRNA3	A0A1Y0DIG3		1.00	< 10e-50	147

a The identity means the same between the interesting sequence and the reference sequence shown in the UniProt ID column, ranging from 0.00 to 1.00, with 1.00 being the highest identity.

b A measure of the significance of the match. The lower the expected value, the better the hit. It depends on the length of the query sequence and the database size. No cut-off is determined.

c The reference sequence's number of amino acid residues matched the interesting sequence

### 3.4 The AAV-delivered CRISPR-Cas9 system with gRNA2 effectively suppressed cccDNA accumulation and HBV replication in d-imHC cells

**Fig. 4:**
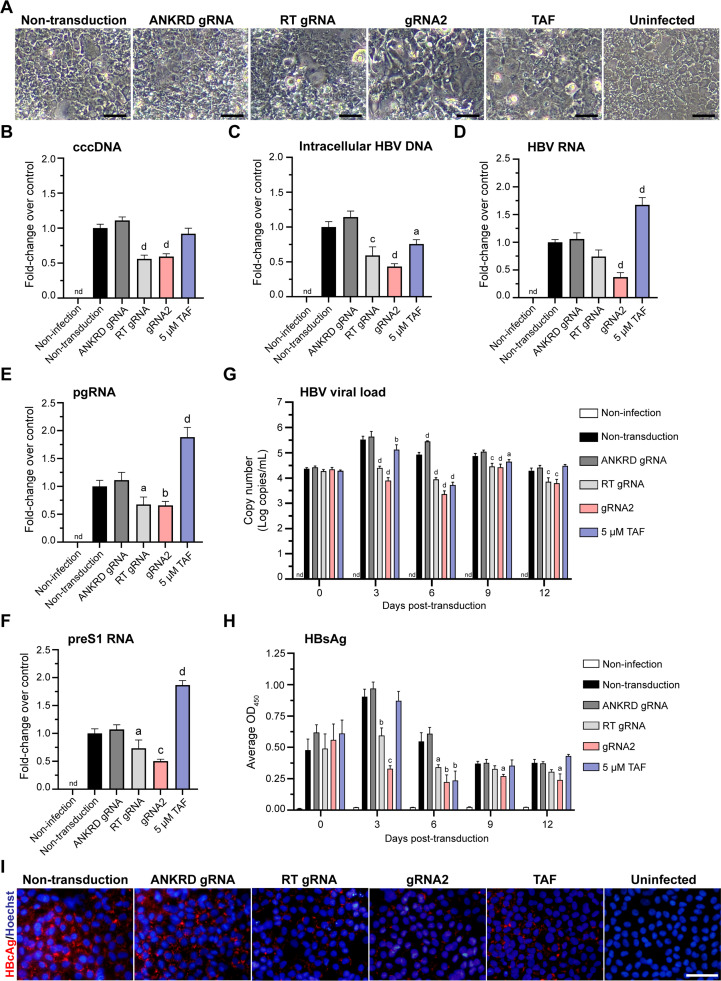
**AAV-delivered CRISPR-Cas9 system with guide RNAs suppresses the HBV life cycle in an *in vitro* HBV infection model.** (**A**) Morphology of d-imHC cells on day 16 post-HBV infection (day 12 post-transduction). Cells were infected with HBV for 3 days, followed by transduction with AAV carrying individual gRNAs for 24 h, and then cultured for an additional 12 days. Tenofovir alafenamide (TAF) was used as a control, added on day 4 post-HBV infection. Scale bars = 50 μm. (**B**) cccDNA and (**C**) intracellular HBV DNA levels in cell pellets were quantified by qPCR, and expressed as fold-change relative to the non-transduced group. (**D**) HBV RNA, (**E**) pgRNA, and (**F**) preS1 RNA levels in cell pellets were measured by qPCR, normalized to GAPDH, and presented as fold change compared with the non-transduced group. (**G**) HBV viral load in the culture supernatant was measured every 3 days by qPCR, using a standard curve generated from a known concentration of HBV 1.3-mer WT replicon plasmid. (**H**) HBsAg levels in the culture supernatant were quantified by ELISA. (**I**) Hepatitis B core antigen (HBcAg) expression was detected in HBV-infected cells following transduction with AAV-CRISPR/Cas9-gRNA2 or treatment with 5 μM TAF, using immunofluorescence assay (IFA) on day 7 post-transduction. Scale bar = 50 μm. The letters (a, b, c, and d) represent statistical differences between the treatments and their respective controls with a *p*-value less than 0.05, 0.01, 0.001, and 0.0001, respectively. ND = not detectable.

The AAV-delivered CRISPR/Cas9 system incorporating gRNA2, which exhibited the strongest anti-HBV activity in HepG2.2.15 cells, was further evaluated in differentiated imHCs (d-imHCs), a physiologically relevant HBV infection model expressing sodium taurocholate co-transporting polypeptide (NTCP) [33]. Following AAV transduction of HBV-infected d-imHCs, multiple markers of HBV replication were assessed over a 12-day period. No discernible changes in cell morphology were observed across treatment groups (Fig. 4A). In addition to morphological assessment, cytotoxicity was evaluated at both cellular and molecular levels following treatment with AAV-delivered CRISPR/Cas9 carrying the designed guide RNAs in imHC cells. No cytotoxic effects were detected by MTT assay (Fig. S3). Furthermore, Annexin V/PI staining revealed no evidence of apoptosis-associated cytotoxicity in treated cells (Fig. S4). Exonuclease-assisted quantitative PCR analysis showed a slight increase in cccDNA levels in cells transduced with ANKRD gRNA (1.11-fold), whereas significant reductions were observed with RT gRNA (0.56-fold) and gRNA2 (0.59-fold). In contrast, tenofovir alafenamide (TAF) treatment resulted in only a modest change (0.92-fold) relative to the control (Fig. 4B). HBV cccDNA levels in cells transduced with RT gRNA and gRNA2 were further validated by ddPCR, revealing an approximate 60% reduction relative to the control (Fig. S5). Consistent with these findings, intracellular HBV DNA levels were increased in the ANKRD group (1.14-fold), substantially reduced in the RT gRNA group (0.59-fold), and markedly suppressed in the gRNA2 group (0.43-fold), outperforming TAF treatment (0.76-fold) (Fig. 4C). Analysis of total HBV RNA at day 12 post-transduction demonstrated minimal suppression in the RT gRNA group (0.74-fold) and substantial inhibition in the gRNA2 group (0.37-fold) (Fig. 4D). Specifically, pregenomic RNA (pgRNA) levels were reduced to 0.67-fold and 0.65-fold in the RT and gRNA2 groups (Fig. 4E), respectively, whereas preS1 RNA, which encodes the large hepatitis B surface protein (L-HBs), was notably suppressed in the gRNA2 group (Fig. 4F). No significant changes were observed in ANKRD gRNA-treated cells, while TAF-treated cells exhibited increased HBV RNA levels (1.6–1.8-fold). Extracellular HBV DNA levels were comparable across all groups at day 0 post-transduction (approximately 4.30 log copies/mL). By day 3, viral loads increased to 5.53 log copies/mL in both non-transduced and ANKRD gRNA-transduced cells, whereas RT gRNA and gRNA2 treatments reduced viral loads to 4.41 and 3.89 log copies/mL, respectively; TAF-treated cells showed a modest reduction to 5.13 log copies/mL (Fig. 4G). By day 6, viral suppression was maintained in the RT gRNA (3.95 log copies/mL), gRNA2 (3.35 log copies/mL), and TAF (3.72 log copies/mL) groups, while viral loads in the ANKRD group continued to increase (5.45 log copies/mL). From day 9 to day 12, sustained suppression was observed in the gRNA2 and RT gRNA groups, whereas viral loads in TAF-treated cells rebounded to levels comparable to non-transduced controls (Fig. 4G). HBsAg levels in culture supernatants were significantly reduced by gRNA2 and RT gRNA as early as day 3 post-transduction (0.33 and 0.59 optical density units (OD), respectively) compared with non-transduced controls (0.90 OD.) (Fig. 4H). While suppression persisted through day 6 in both groups, only gRNA2 maintained significantly lower HBsAg levels through day 12. Treatment with ANKRD gRNA had no significant effect at any time point (Fig. 4H). HBV core antigen (HBcAg) expression was assessed by immunofluorescence assay at day 7 post-transduction (Fig. 4I). Strong HBcAg fluorescence signals were observed in non-transduced cells and in cells transduced with ANKRD gRNA. In contrast, fluorescence intensity was reduced by approximately 50% in TAF-treated cells and was largely absent in cells treated with RT gRNA or gRNA2, indicating effective suppression of HBV core protein expression. Collectively, these findings demonstrate that AAV-mediated delivery of CRISPR/Cas9 with gRNA2 effectively suppresses HBV replication in the infection model by reducing cccDNA levels, intracellular viral DNA, transcriptional activity, and virion production, without inducing cytotoxicity.

### 3.5 On-target activity of CRISPR-Cas9 carrying gRNA2

**Fig. 5:**
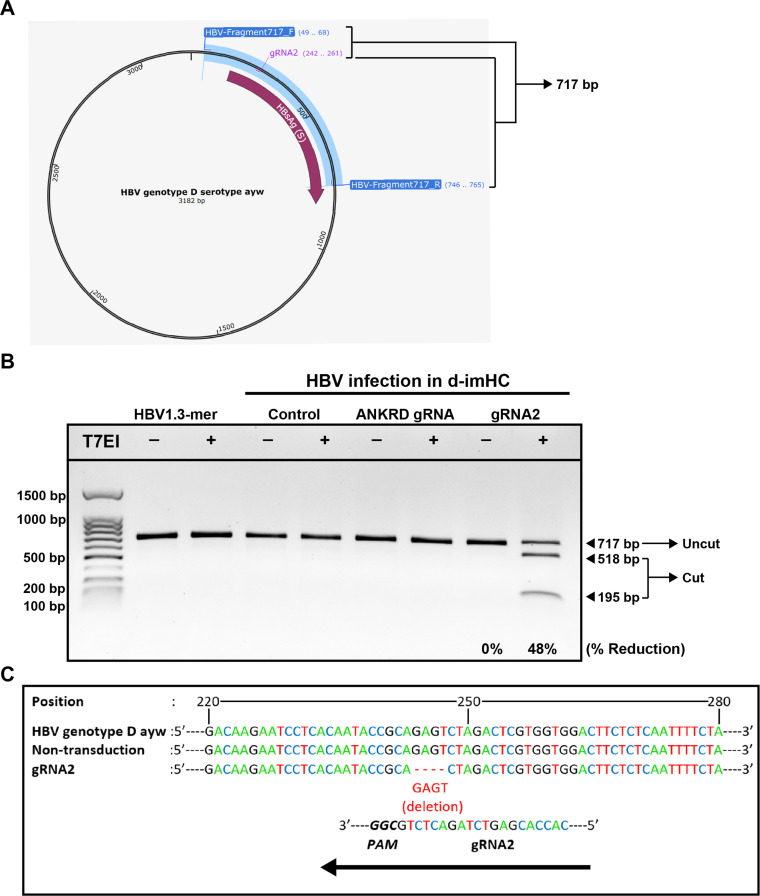
**Analysis of the mispaired HBV genomic DNA via T7E1 assay.** DNA sequence of HBV genotype D serotype *ayw* was used as template to design primer pairs for covering the CRISPR/Cas9 target site of gRNA2. (**A**) The HBV genomic DNA map presents the position of specific primer binding sites, generating PCR amplicon 717 bp. (**B**) Analysis of the mispaired HBV genomic DNA by T7E1 assay. The specific primer pairs for covering the CRISPR/Cas9 target site of gRNA2 can amplified PCR fragment from HBV1.3-mer WT replicon plasmid and HBV genomic DNA extracted from HBV-infected cells with AAV transduction. The PCR fragments in all conditions were treated with or without T7E1, and were further analyzed by gel-electrophoresis. The intensity of DNA bands was also measured and presented as % reduction from uncut DNA fragment. Targeted sequencing confirmed a specific indel event, consisting of a four-nucleotide “GAGT” deletion at the gRNA2-directed CRISPR/Cas9 cleavage site in HBV-infected imHC cells (**C**).

Double-strand breaks (DSBs) induced by CRISPR/Cas9 are predominantly repaired through the error-prone non-homologous end joining (NHEJ) pathway, resulting in insertions or deletions (indels) at the target site. To assess on-target genome editing by gRNA2, HBV target sequences were analyzed using a T7 endonuclease I (T7E1) mismatch cleavage assay. The HBV genotype D, serotype *ayw* reference sequence (GenBank accession no. U95551.1) was used to design primer pairs flanking the gRNA2 target site (Fig. 5A). The forward primer annealed to the antisense strand at nucleotide positions 49–68, and the reverse primer annealed to the sense strand at positions 746–765, yielding a 717-bp amplicon. These primer pairs successfully amplified the target region from the HBV 1.3-mer wild-type replicon plasmid, which served as a positive control for PCR amplification, as well as from genomic HBV DNA extracted from HBV-infected cells following AAV transduction with ANKRD gRNA or gRNA2 (Fig. 5B). PCR products from each condition were subjected to denaturation and reannealing to allow heteroduplex formation, followed by treatment with or without T7E1 before analysis by agarose gel electrophoresis. PCR amplicons derived from cells transduced with CRISPR/Cas9 carrying gRNA2 were efficiently cleaved by T7E1 into fragments of approximately 195 bp and 518 bp, accompanied by a 48% reduction in the intensity of the full-length amplicon, indicating the presence of heteroduplex DNA generated by indel formation (Fig. 5B). In contrast, PCR products from control conditions, including ANKRD gRNA-transduced cells, showed no detectable cleavage following T7E1 treatment. These cleavage patterns reflect NHEJ-mediated repair events subsequent to CRISPR/Cas9-induced double-strand breaks in the HBV genome mediated by gRNA2. Sanger sequencing of PCR amplicons spanning the excision site (Fig. 5C) revealed that gRNA2-guided CRISPR/Cas9 cleavage followed by NHEJ repair resulted in a four-nucleotide “GAGT” deletion at the target site in HBV genome. Collectively, these results provide evidence of specific on-target activity of gRNA2 within the HBV genome.

## 4 Discussion

Chronic HBV infection remains a significant clinical challenge due to the persistence of covalently closed circular DNA (cccDNA) within infected hepatocytes. In recent years, the CRISPR/Cas9 system has emerged as a promising approach for functional inactivation of cccDNA. Guide RNAs (gRNAs) have been designed to target conserved regions [[21], [22], [23], [24]], to optimize predicted on-target efficiency while minimizing off-target potential [21,27,29], or to disrupt essential viral genes such as HBx [25]. In this study, we designed gRNAs targeting the overlapping region between the early open reading frame (ORF) of the HBV surface antigen and the central ORF of the DNA polymerase gene. This region was selected for its high sequence conservation across HBV genotypes A–J, moderate predicted cleavage efficiency, and low off-target potential in both the HBV and human genomes. Although these gRNAs do not discriminate between cccDNA and relaxed circular DNA (rcDNA), largely due to the limited availability of suitable PAM sites, their effectiveness is supported by the coexistence of multiple HBV DNA forms within infected cells, thereby increasing the likelihood of CRISPR/Cas9-mediated disruption. The Cas9 gene and gRNAs were delivered via adeno-associated virus (AAV) vectors with high hepatocyte tropism [45]. Previous work using AAV vectors in similar cell systems reported minimal cytotoxicity at day 6 post-transduction [21]. Consistent with these observations, no morphological alterations were detected in HepG2.2.15 cell. Accordingly, assessment of additional replicative intermediates, including intracellular HBV DNA, is necessary to confirm suppression of virion production. The observed reduction in intracellular HBV DNA levels further supports the efficacy of CRISPR/Cas9 treatment. Previous research has shown that inhibition of HBV DNA levels in HepG2.2.15 cells depends on the multiplicity of genome (MOG) of the AAV vector used, ranging from 2 × 10⁴ to 2 × 10⁶ [21]. Other studies have reported 10–125-fold reductions in intracellular HBV DNA, depending on the gRNA target region (e.g., core, surface antigen, or reverse transcriptase “YMDD” motif) [26]. Extracellular HBV DNA, representing secreted mature virions, was also measured to assess the effects of CRISPR/Cas9 treatment. The rise in HBV viral load in untreated HepG2.2.15 cells is attributable to intracellular amplification, as these cells do not express NTCP and therefore cannot support reinfection [46]. One study reported a 32.1% reduction at day 3, slightly decreasing to 30.3% at day 6 [21]. In contrast, others showed over 50% reduction at day 3 [47], 100–800-fold reduction compared to non-specific gRNA controls [26], and 80–90% reduction by day 9 compared to gRNA-empty controls [48]. Similarly, our study demonstrated a >100-fold reduction in extracellular HBV DNA by day 12, highlighting the potent antiviral effect of our CRISPR/Cas9 approach. The relationship between HBV markers and HBsAg levels remains correlated in our study. Like, most studies have found a correlation between HBsAg, cccDNA, extracellular HBV DNA, and HBeAg levels [[48], [49], [50]]. Kostyushev et al. reported discordant expression levels in CRISPR/Cas9-treated HepG2 cells using gRNA Sp40, with low S-RNA but moderate HBsAg levels [27]. Other investigations have shown that HBsAg levels initially decrease following treatment, only to rebound later [21,48], even as other HBV DNA markers remain suppressed. These discrepancies could be due to differences in gRNA target regions, delivery systems (ribonucleoproteins, plasmids, or viral vectors), and cell models (e.g., HepG2.2.15, HepG2-NTCP, HepaRG, HepAD38). In our study, HBsAg levels decreased moderately in HepG2.2.15 cells, but showed a substantial reduction in naturally infected d-imHC cells, despite marked reductions in other HBV markers. One possible explanation is that integrated HBV DNA, rather than cccDNA, is the primary source of HBsAg in hepatoma cell lines such as HepG2.2.15. A study by Wang et al. demonstrated that serum HBsAg did not correlate with intrahepatic cccDNA in hepatocellular carcinoma (HCC) patients, suggesting that integrated HBV DNA was the primary source of HBsAg production [51]. Our findings support this hypothesis, as HBsAg was only modestly affected by CRISPR/Cas9-mediated cccDNA disruption. While HepG2.2.15 cell line is suitable for evaluating antiviral activity against HBV replication, it does not represent the full viral life cycle. Importantly, HepG2.2.15 cells harbor HBV DNA integrated into the host genome, which limits their suitability for evaluating antiviral activity against cccDNA [46]. Characterization of their anti-HBV efficacy after CRISPR/Cas9 intervention in a physiologically relevant infection model is necessary. The immortalized hepatocyte-like cell line (imHC), derived from human bone marrow mesenchymal stem cells (hMSCs), exhibits key hepatocyte phenotypes [32] and supports infection by a range of human hepatotropic pathogens, including *Plasmodium vivax* [52], Hepatitis C virus (HCV) [35], Dengue virus [53], and Hepatitis B virus (HBV) [33]. The imHC cell line has also been employed as an *in vitro* model for evaluating anti-HBV activity of herbal extracts, phytochemicals, and antiviral agents [33,34,39,54]. Therefore, the imHC cell line represents a suitable *in vitro* model for evaluating the efficacy of CRISPR/Cas9 against HBV infection. Consistent with findings in HepG2.2.15 cells, no morphological alterations were observed in HBV-infected imHC cells up to 12 days post-transduction. Furthermore, AAV-mediated delivery of CRISPR/Cas9 induced no detectable cytotoxic effects at either the cellular or molecular levels, suggesting that the designed guide RNAs did not produce significant off-target cytotoxicity. Using this infection model, evaluation of additional replicative intermediates, including HBV DNA and RNA, is important for validating the efficiency of RT gRNA and gRNA2. The newly designed gRNA2 demonstrated superior antiviral efficacy compared with RT gRNA by reducing HBV DNA and multiple HBV RNA transcripts, including total RNA, pgRNA, and PreS1 RNA. Then, gRNA2 treatment resulted in a >100-fold reduction in extracellular HBV DNA as well as a >60% decrease in HBsAg levels at day 6, highlighting the potent antiviral activity of this strategy. Using this infection model, evaluation of cccDNA levels is essential for validating the antiviral efficacy of gRNA2. The cccDNA levels were quantified by exonuclease-assisted quantitative PCR to assess antiviral efficacy [33,34]. The results showed that both the RT gRNA and the newly designed gRNA2 significantly reduced cccDNA levels by up to 50%. Furthermore, HBV cccDNA levels in cells transduced with RT gRNA or gRNA2 were validated by droplet digital PCR (ddPCR) to enhance specificity and sensitivity [39], revealing an approximate 60% reduction. Previous studies have reported variable cccDNA reductions, ranging from 16–27% [21] to 60–75% [22], within days after CRISPR/Cas9 treatment. The cccDNA formation has also been suppressed at ∼50% by day 6 [21,27], and up to a 10-fold reduction by day 14 [26]. Thus, the newly designed gRNA2-mediated CRISPR/Cas9 system effectively reduced cccDNA levels and suppressed HBV infection *in vitro*, although complete disruption of cccDNA was not achieved. However, the significance of these findings is limited by the absence of *in vivo* studies to further validate the anti-HBV efficacy of the selected gRNA for CRISPR/Cas9 approach.

To assess CRISPR/Cas9 activity, Sanger sequencing was primarily used as a preliminary method to detect editing events at the target sites. Although this approach is less sensitive and less quantitative than next-generation sequencing (NGS) or methods such as IDAA and TIDE [55,56], it remains useful for identifying sequence disruption and indel formation associated with CRISPR/Cas9-mediated DNA cleavage. In the present study, sequencing analysis of gRNA-treated samples demonstrated chromatogram disruption and mixed sequencing signals near the predicted Cas9 cleavage regions, consistent with heterogeneous indel formation generated through non-homologous end joining (NHEJ)-mediated repair [57]. In contrast, the non-transduced control samples maintained intact wild-type sequence continuity across the target region. Previous studies have shown that deletions are more common than insertions and can range from 1 to over 10 nucleotides [22,[25], [26], [27],^29^,^30^,^48^,^58^,^59^]. Although PAM site deletions have been reported [22,25,26], we did not observe clear evidence of PAM deletion in the present study. Because the sequencing analysis was performed using bulk cell populations rather than single-cell clones, the resulting chromatograms likely represented mixed edited and unedited viral genomes with variable indel frequencies. Therefore, precise characterization of individual indel patterns and exact deletion sizes could not be determined with high confidence from the current Sanger sequencing data alone. This limitation may also contribute to the similar inhibition levels observed among different gRNAs. Accordingly, the predicted mutations and downstream protein structural analyses should be interpreted cautiously as supportive assessments of potential functional disruption rather than definitive characterization of all edited variants. Future studies using targeted deep sequencing or clonal analysis will be necessary to more accurately define indel composition, editing frequency, and on-target/off-target editing outcomes. CRISPR/Cas-induced DNA double-strand breaks (DSBs) are frequently repaired through the error-prone NHEJ pathway, leading to the formation of insertions and deletions (indels) [57]. To further support the presence of CRISPR/Cas9-mediated mutations, target regions within the HBV genomic DNA were additionally analyzed using a T7 endonuclease I (T7E1) mismatch cleavage assay, which detects sequence mismatches resulting from indel formation [22].

In a physiologically relevant HBV infection model (d-imHC cells), the AAV-delivered CRISPR/Cas9 system carrying gRNA2 demonstrated potent anti-HBV activity. gRNA2 significantly reduced cccDNA levels (0.59-fold), outperforming TAF (0.92-fold), and strongly suppressed intracellular HBV DNA (0.43-fold vs. TAF’s 0.76-fold). Viral RNA transcripts, including total HBV RNA, pgRNA, and preS1 RNA, were markedly inhibited, with gRNA2 showing the greatest reductions. Viral loads and HBsAg secretion were consistently lower in gRNA2-treated cells compared with other groups throughout the 12-day observation period, with sustained suppression superior to TAF, which showed partial viral rebound. Immunofluorescence analysis also confirmed strong inhibition of HBcAg expression by gRNA2. Collectively, these findings support that AAV-delivered CRISPR/Cas9-gRNA2 system effectively suppresses multiple aspects of HBV replication, including cccDNA persistence, viral transcription, and viral protein expression in d-imHC cells.

## 5 Conclusions

Targeting HBV at the overlapping ORFs of HBsAg and Pol is a possible CRISPR/Cas9 strategy. Specifically, when delivered by AAV vectors, CRISPR/Cas9 with gRNA2 efficiently disrupted HBV DNA, reduced cccDNA accumulation, and suppressed viral replication, most likely by compromising intracellular amplification pathways. This approach warrants further investigation for therapeutic applications.

## Declaration of generative AI and AI-assisted technologies in the manuscript preparation process

During the preparation of this manuscript, the authors used ChatGPT-5 to improve language and readability. After using this tool, the authors reviewed and edited the content as needed and take full responsibility for the content of the published article.

## Funding

This research paper is supported by Specific League Funds from Mahidol University. This work was financially supported by Reinventing University System, Drug discovery platform, Mahidol University awarded to K. Sa-ngiamsuntorn. This research project is supported by Mahidol University, the Ramathibodi Foundation (Children Cancer Project), the Science Achievement Scholarship of Thailand (SAST), and the Fundamental Fund from Thailand Science Research and Innovation (FRB650007/0185).

## CRediT authorship contribution statement

**Pattida Kongsomboonchoke:** Writing – original draft, Validation, Resources, Investigation, Data curation, Conceptualization. **Yongyut Pewkliang:** Writing – original draft, Visualization, Validation, Methodology, Formal analysis, Data curation. **Piyanoot Thongsri:** Visualization, Validation, Methodology, Investigation. **Alisa Tubsuwan:** Validation, Investigation, Data curation, Conceptualization. **Kanit Bhukhai:** Methodology, Data curation, Conceptualization. **Nithi Asavapanumas:** Visualization, Data curation, Conceptualization. **Phetcharat Phanthong:** Investigation, Data curation, Conceptualization. **Suparerk Borwornpinyo:** Data curation, Conceptualization. **Wararat Chiangjong:** Writing – original draft, Supervision, Methodology, Investigation, Formal analysis, Data curation, Conceptualization. **Khanit Sa-ngiamsuntorn:** Writing – review & editing, Writing – original draft, Supervision, Resources, Project administration, Investigation, Funding acquisition, Conceptualization. **Suradej Hongeng:** Validation, Supervision, Resources, Project administration, Funding acquisition, Data curation, Conceptualization.

## Declaration of competing interest

The authors declare that they have no known competing financial interests or personal relationships that could have appeared to influence the work reported in this paper.

## Data Availability

All data generated and analyzed in this study are provided in the article and its supplementary materials.

## Appendix B Supplementary materials

Image, application 1

Image, application 2

Image, application 3

Image, application 4

Image, application 5

Image, application 6

Image, application 7
