# Supplementary figures and images for: AAV-mediated delivery of CRISPR/Cas9 targeting conserved overlapping ORFs efficiently suppresses HBV replication in hepatocyte models
Source: Biotechnol Rep (Amst). 2026 May 19;51:e00961. doi: 10.1016/j.btre.2026.e00961 (PMC13234202; doi:10.1016/j.btre.2026.e00961)

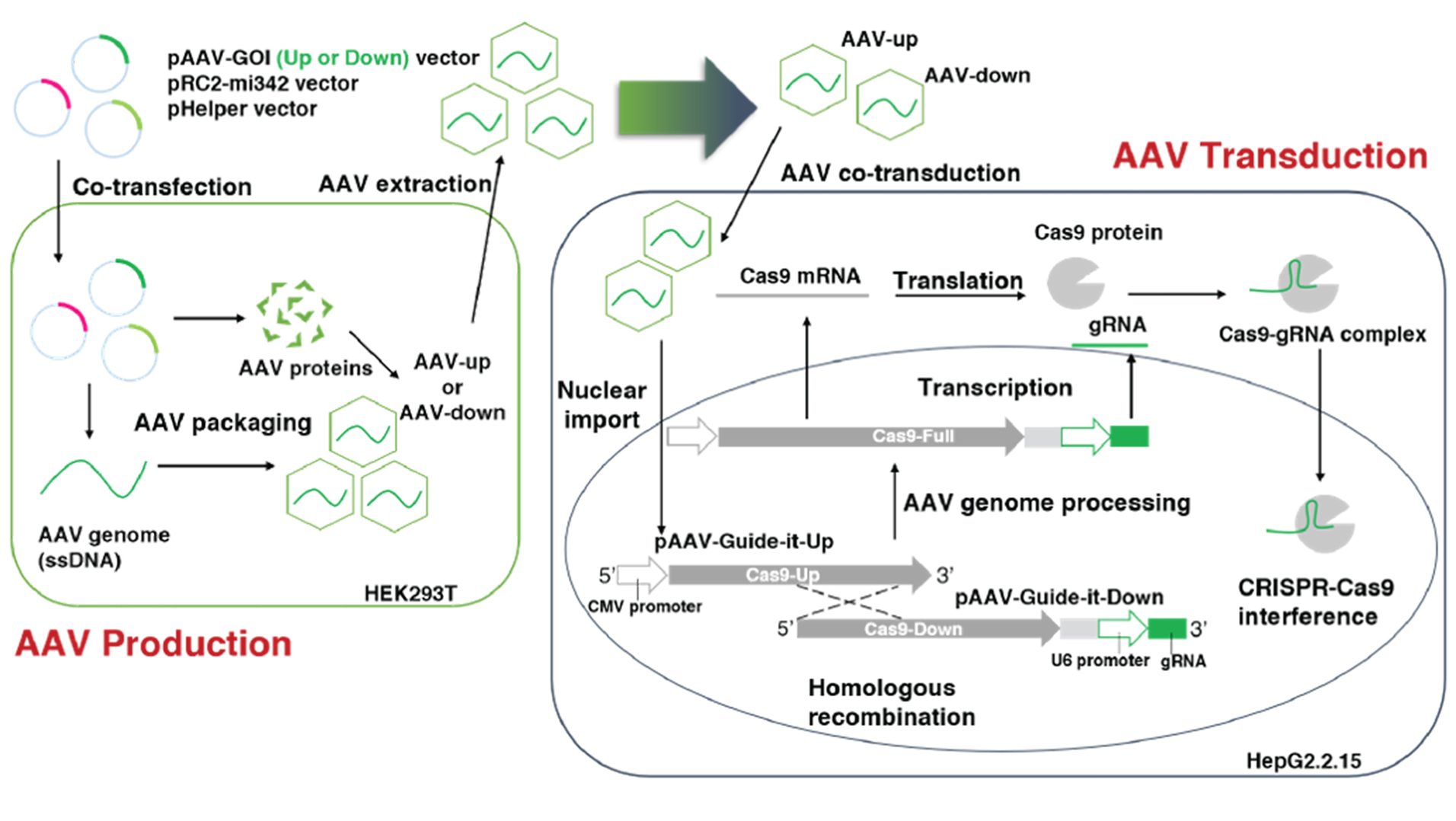

Supplement: Supplementary file 1 [file mmc1.zip › Figure S1.tif]

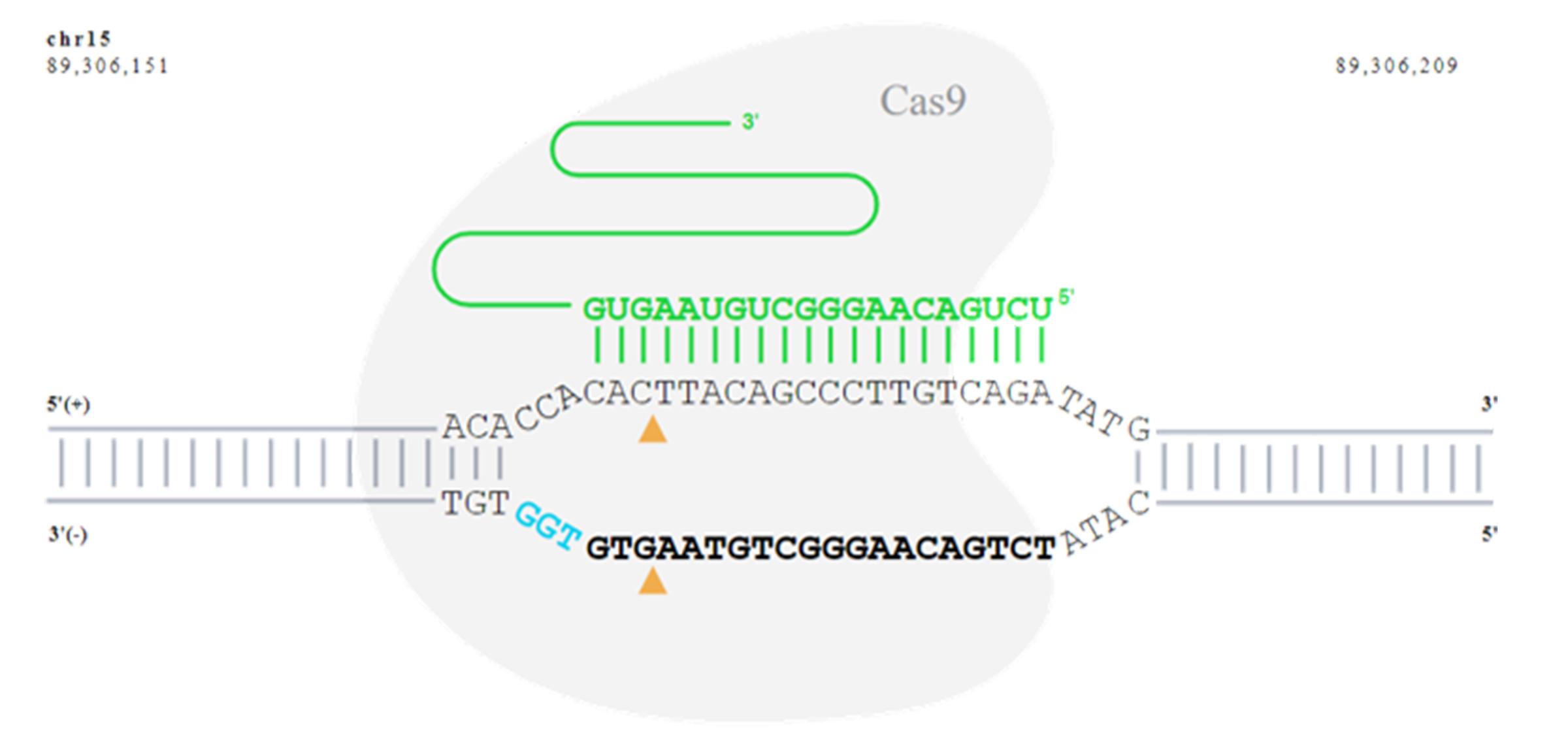

Supplement: Supplementary file 2 [file mmc2.zip › Figure S2.tif]

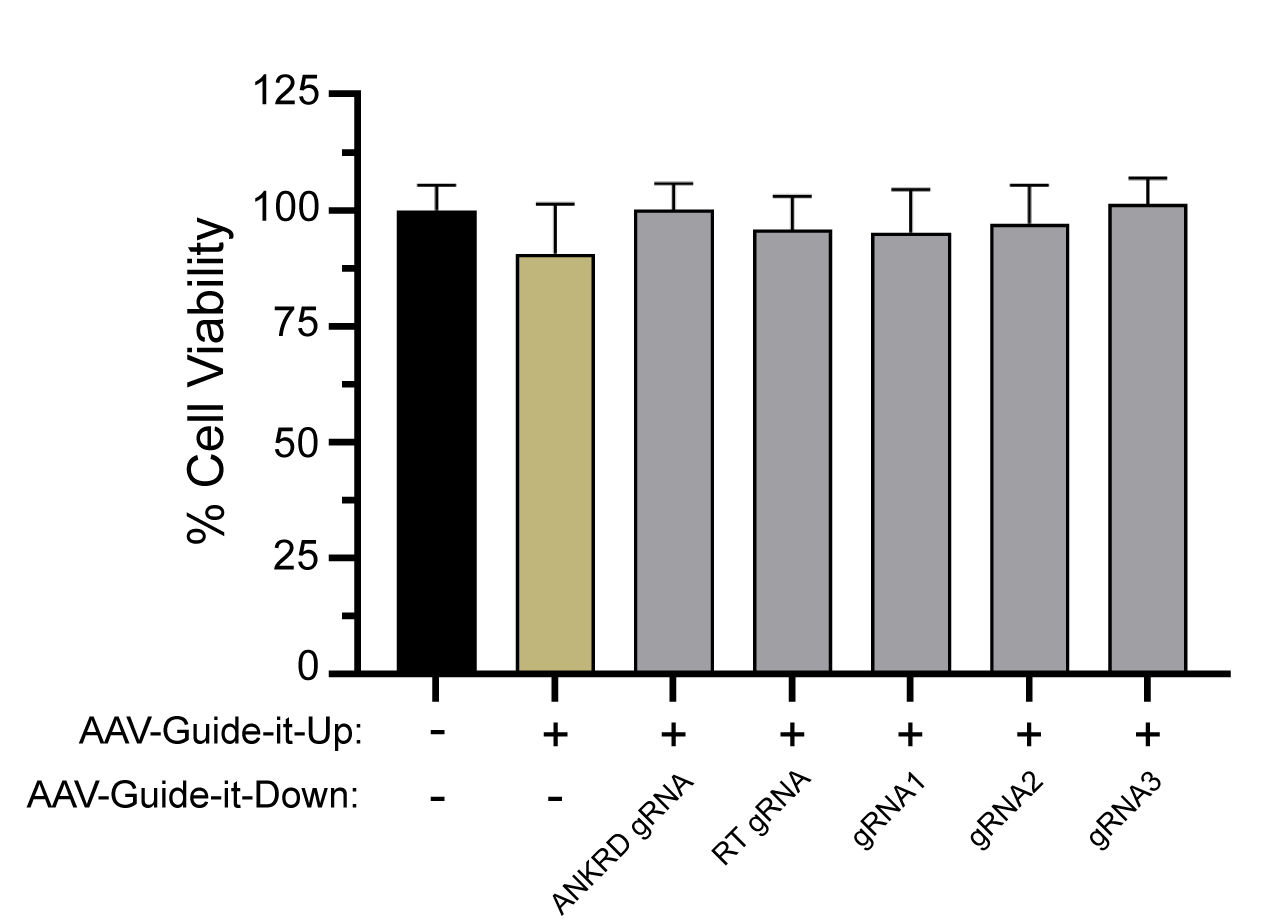

Supplement: Supplementary file 3 [file mmc3.zip › Figure S3.tif]

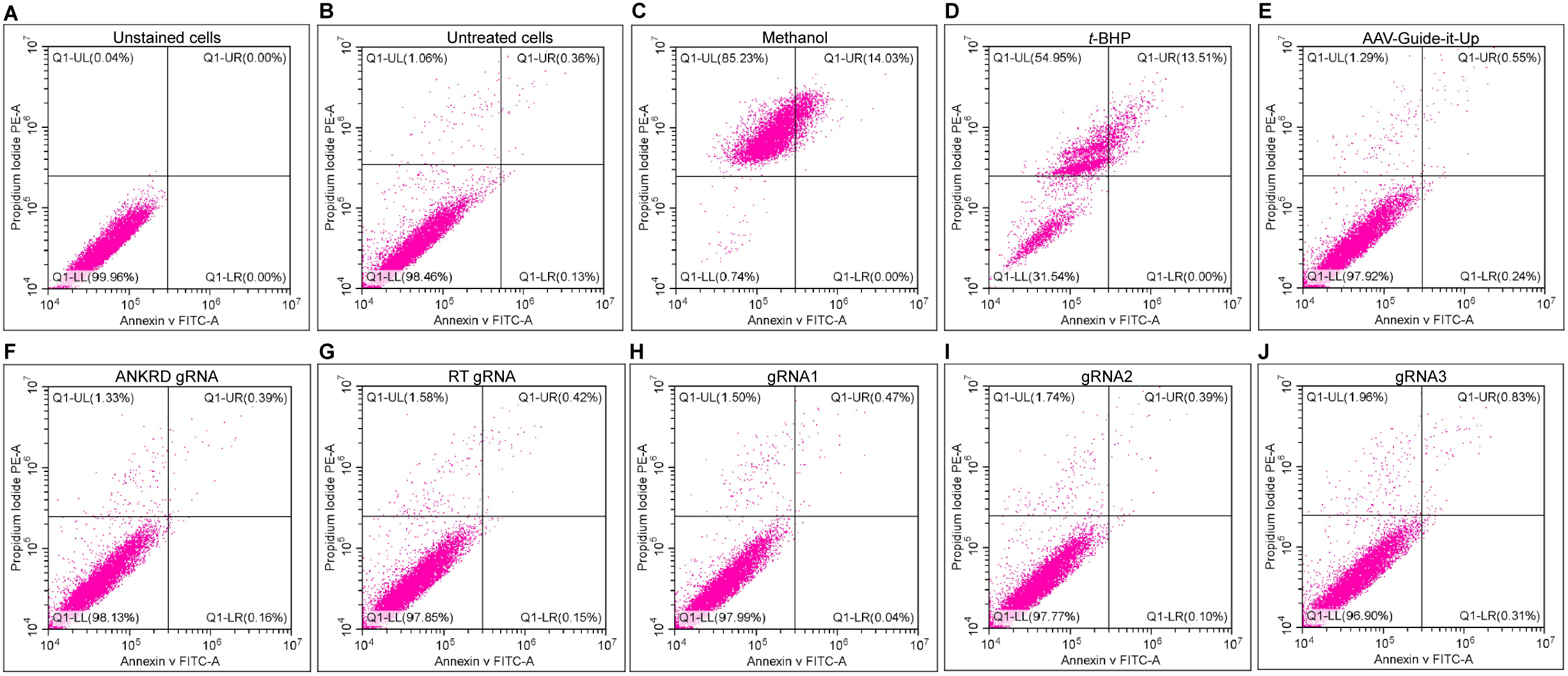

Supplement: Supplementary file 4 [file mmc4.zip › Figure S4.tif]

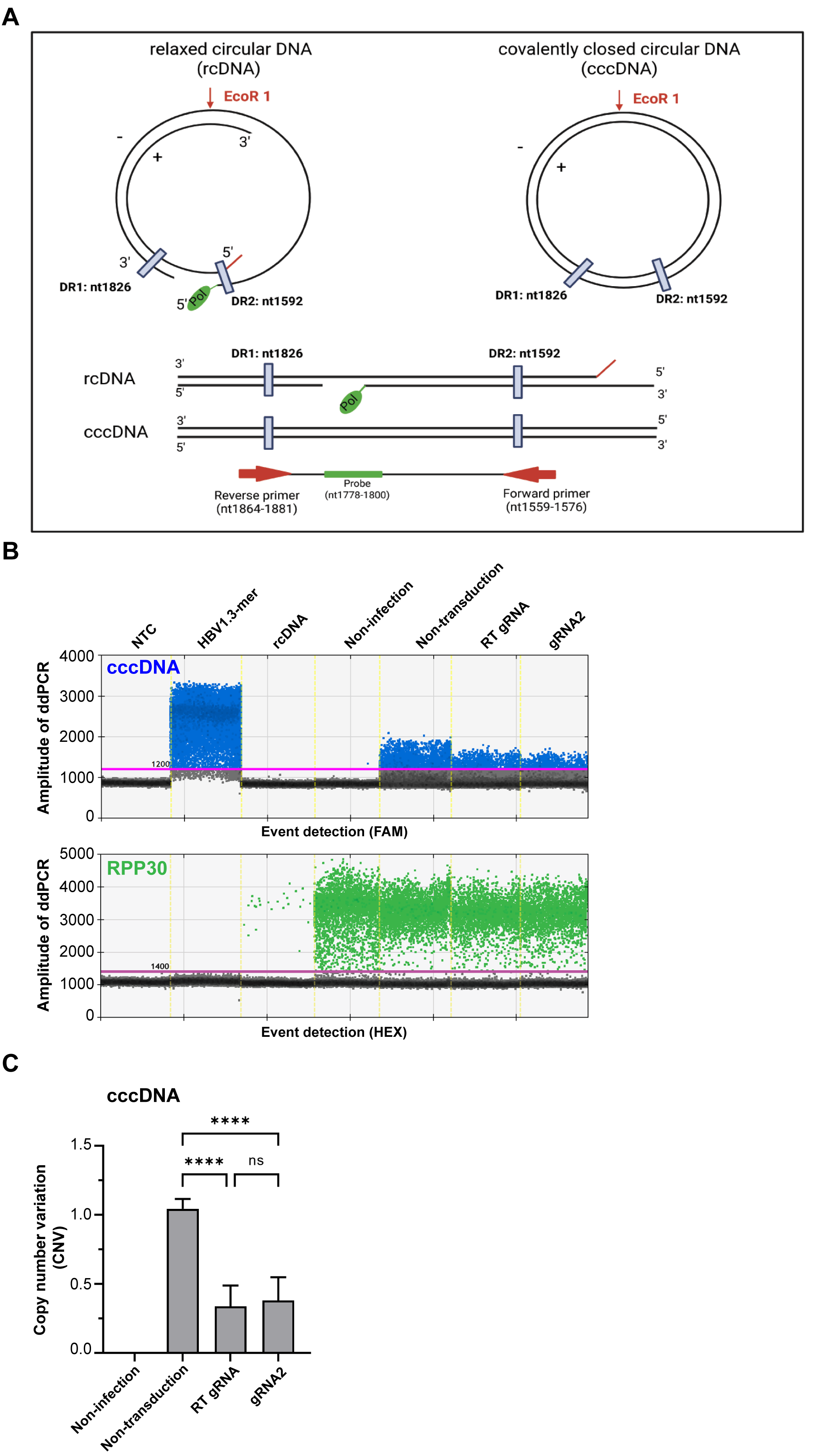

Supplement: Supplementary file 5 [file mmc5.zip › Figure S5.tif]
