# Supplementary material for: AAV-mediated delivery of CRISPR/Cas9 targeting conserved overlapping ORFs efficiently suppresses HBV replication in hepatocyte models
Source: Biotechnol Rep (Amst). 2026 May 19;51:e00961. doi: 10.1016/j.btre.2026.e00961 (PMC13234202; doi:10.1016/j.btre.2026.e00961)
Supplement: Supplementary file 6 [file mmc6.pdf]

Order No: HC01459148 File: Sample_2_F_HepG2_2_15_HBV-717-F.ab1 Run Ended: 2026/5/6 17:39:52 Signal G:49 A:81 C:135 T:98

Sample: Sample_2_F_HepG2_2_15_HBV-717-F Lane: 35 Base spacing: 14.9400425 692 bases in 12695 scans

10 20 30 40 50 60 70 80 90 100 110 120

T GGG GGGGGCG AACC GGG GT GGAG AA ACCC TAC CAACCACTG AACAAAT GGCACT GGT AAACTGAGCC GTG AGAAACGGGCTGAGGCCCT CTCCCCTAGGAATTTTCCGAAAGCCCACGAGGA

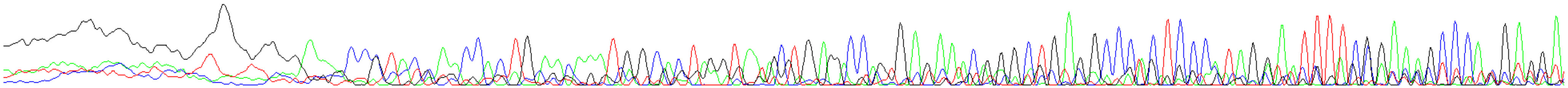

130 140 150 160 170 180 190 200 210 220 230 240

TGGGATGGGAATACAGGTGCAATTTCCGTCCGAAGGTTTGGTACAGCAACAAGAGGGATACAGAAAGGTTCCTTGATCAA TA GTCATGC T GTTC GGCATGGTCCCGTGCTGGTGGTTGAGGAT

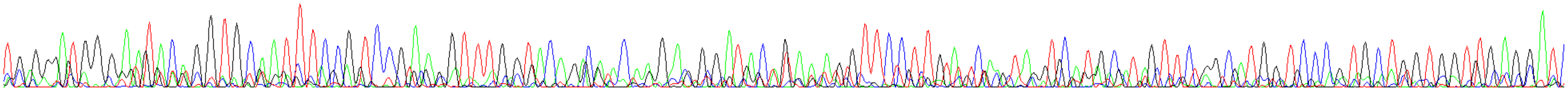

250 260 270 280 290 300 310 320 330 340 350 360 370

CCTGGAATTAAAGGACAAACTGCCGACATACCTTGATGGTCCGGAATAACCAACAGGAAGATGAG GCATAG CAGCACGATGAAGAGGAAGATGATAAAACGCCGCA CACACATCCGGCGATAACCAG

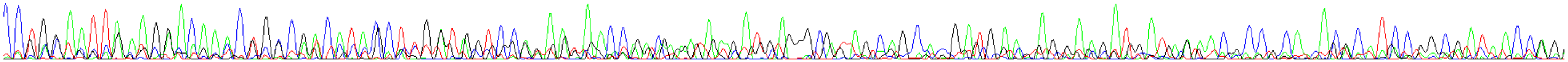

380 390 400 410 420 430 440 450 460 470 480 490

GACAAGTTGGAGGACAGGAGGTTGGT GATGATGGTTTGG GTGC AGCAACCGACTTTTTGGGACAATGGT TAAATTGC TGACTA TAGGGCGGGGGAGGTCCACGCCT AGGCCCTGTTGTTCTTTT

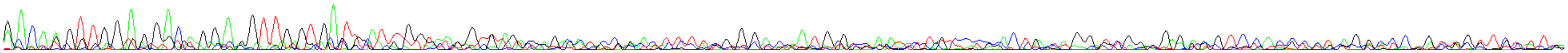

500 510 520 530 540 550 560 570 580 590 600 610 620

ATGC CT TTGGACG G AT TCTCA CAAGAATGCCCTTCCCTGGACAACGGGGAGGCGAAG GATTCG GAA AGGAATGGGCTCC GGCC CTTT GCG GAGGGCCGGCTT CTC AG GGACACC TTCAAGGCT

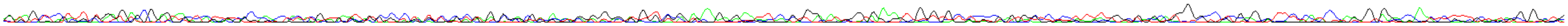

Order No: HC01459148   File: Sample_2_F_HepG2_2_15_HBV-717-F.ab1   Run Ended: 2026/5/6 17:39:52   Signal G:49 A:81 C:135 T:98

Sample: Sample_2_F_HepG2_2_15_HBV-717-F   Lane: 35   Base spacing: 14.9400425   692 bases in 12695 scans   Page 2 of 2

630 640 650 660 670 680 690

GCTTTGGAGGGCTTTGTGACCTGGTTTGGTGCTACCTTTAAGTTGATGCTGGAGATTTTAGGGACAAAGAA

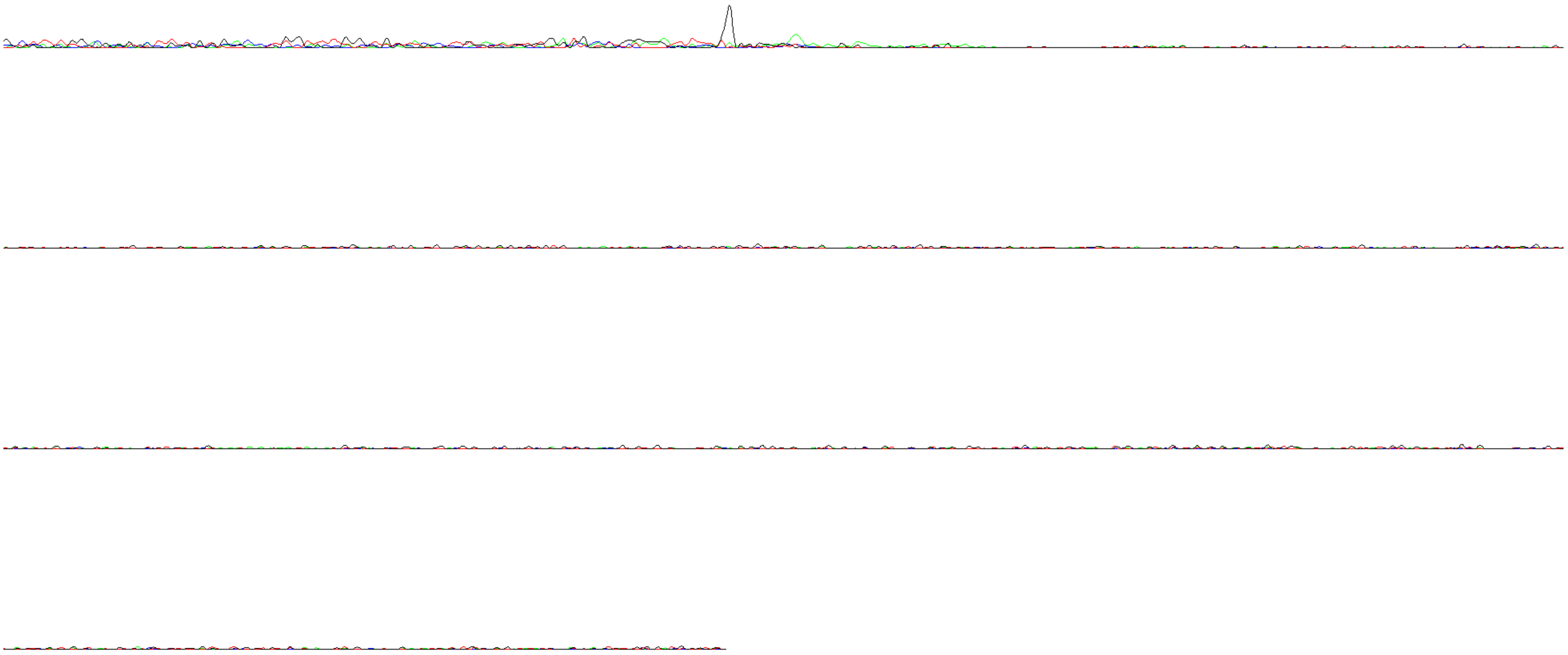

Order No: HC01459148 File: Sample_2_R_HepG2_2_15_HBV-717-R.ab1 Run Ended: 2026/5/6 17:39:52 Signal G:2612 A:6438 C:7087 T:4867

Sample: Sample_2_R_HepG2_2_15_HBV-717-R Lane: 33 Base spacing: 14.907304 1041 bases in 12684 scans Page 1 of 2

TAAGCCAACGTGGGGGAAGCCCTACGAACCACTGAACAAATGGCACTAGTAAACTGAGCCAGGAGAAACGGGCTGAGGCCCACTCCCATAGGAATTTTCCGAAAGCCCAGGATGAT

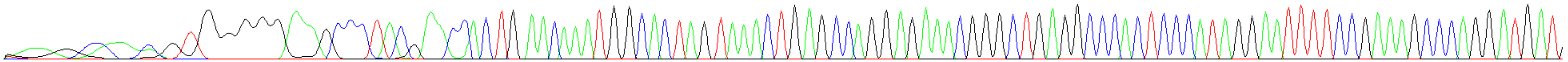

GGGATGGGAATACAGGTGCAATTTCCGTCCGAAGGTTTGGTACAGCAACAGGAGGGATACATAGAGGTTCCTTGAGCAGTAGTCATGCAGGTTCGGCATGGTCCCGTGCTGGTGGTTGAGGATC

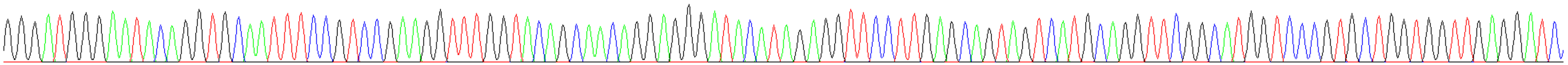

CTGGAATTAGAGGACAAACGGGCAACATACCTTGATAGTCCAGAAGAACCAACAAGAAGATGAGGCATAGCAGCAGGATGAAGAGGAAGATGATAAAACGCCGCAGACACATCCAGCGATAACCAG

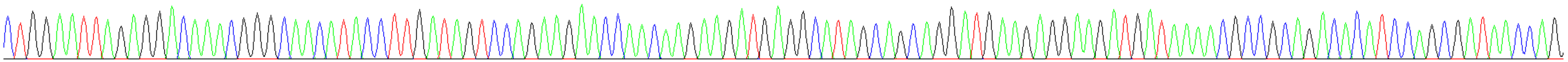

GACAAGTTGGAGGACAGGAGGTTGGTGAGGATTGGAAGGTTGGGGACTGCGAATTTTGGCCAAGACACACGGTAGTTCCCCCTAGAAAATTGAGAGAAGTCCACCACGAGTCTAGACTCTGCGGT

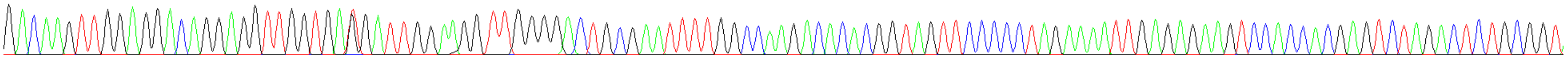

ATTGTGAGGATTCTTGTCAACAAGAAAAACCCCGCCTGTAACACGAGAAGGGGTCCTAGGAATCCTGATGTGATGTTCTCCATGTTCAGCGCAGGGTCCCCAATCCTCGAGAAGATTGACGAT

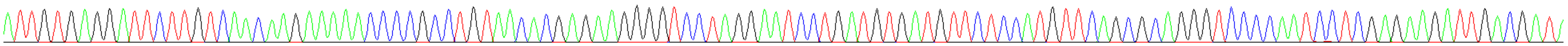

Order No: HC01459148   *File: Sample_2_R_HepG2_2_15_HBV-717-R.ab1*   *Run Ended: 2026/5/6 17:39:52*   *Signal G:2612 A:6438 C:7087 T:4867*

Sample: Sample_2_R_HepG2_2_15_HBV-717-R   *Lane: 33*   *Base spacing: 14.907304*   *1041 bases in 12684 scans*

620 630 640 650 660 670 680 690 700 710 720 730

AAGGGAGAGGCAGTAGTCGGAACAGGGTTTACTGCTCCTGAACTGGAGCCACCACGGGGT AAAAAAAA A A GG GAAAGGGGGGGGGGGGGGGGGGGGGGGGGGGGGGGGGGGGGGGGGGGG

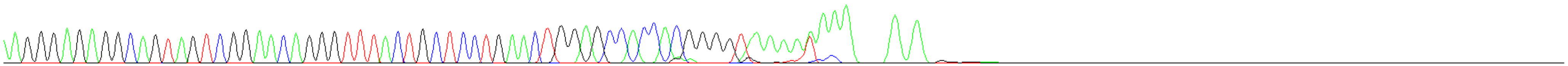

740 750 760 770 780 790 800 810 820 830 840 850

GGGGGGGGGGGGGGGGGGGGGGGGGGGGGGGGGGGGGGGGGGGGGGGGGGGGGGGGGGGGGGGGGGGGGGGGGGGGGGGGGGGGGGGGGGGGGGGGGGGGGGGGGGGGGGGGGGGGGGGGGGGG

860 870 880 890 900 910 920 930 940 950 960 970 980

GGGGGGGGGGGGGGGGGGGGGGGGGGGGGGGGGGGGGGGGGGGGGGGGGGGGGGGGGGGGGGGGGGGGGGGGGGGGGGGGGGGGGGGGGGGGGGGGGGGGGGGGGGG GGGGGGGGGGGGGGGGG

990 1000 1010 1020 1030 1040

GGGGGGGGGGGGGGGGGGGGGGGGGGGGGGGGGGGGGGGGGGGGGGGGGGGGGGGGT
