# Supplementary material for: AAV-mediated delivery of CRISPR/Cas9 targeting conserved overlapping ORFs efficiently suppresses HBV replication in hepatocyte models
Source: Biotechnol Rep (Amst). 2026 May 19;51:e00961. doi: 10.1016/j.btre.2026.e00961 (PMC13234202; doi:10.1016/j.btre.2026.e00961)
Supplement: Supplementary file 7 [file mmc7.docx]

**AAV-mediated delivery of CRISPR/Cas9 targeting conserved overlapping ORFs efficiently suppresses HBV replication in hepatocyte models**

Pattida Kongsomboonchoke^1#^, Yongyut Pewkliang^2#^, Piyanoot Thongsri^2^, Alisa Tubsuwan^3^, Kanit Bhukhai^4^, Nithi Asavapanumas^5^, Phetcharat Phanthong^6^, Suparerk Borwornpinyo^1,7^, Wararat Chiangjong^8^, Khanit Sa-ngiamsuntorn^9,10*^, Suradej Hongeng^2^

^1^Department of Biotechnology, Faculty of Science, Mahidol University, Bangkok, 10400, Thailand

^2^Department of Pediatrics, Faculty of Medicine Ramathibodi Hospital, Mahidol University, Bangkok, 10400, Thailand.

^3^Institute of Molecular Biosciences, Mahidol University, Nakhon Pathom, 73170, Thailand

^4^Department of Physiology, Faculty of Science, Mahidol University, Bangkok, 10400, Thailand

^5^Chakri Naruebodindra Medical Institute, Faculty of Medicine Ramathibodi Hospital, Mahidol University, Samutprakarn, 10540, Thailand

^6^Department of Anatomy, Faculty of Science, Mahidol University, Bangkok, 10400, Thailand

^7^Excellence Center for Drug Discovery, Faculty of Science, Mahidol University, Bangkok, 10400, Thailand

^8^Pediatric Translational Research Unit, Department of Pediatrics, Faculty of Medicine Ramathibodi Hospital, Mahidol University, Bangkok, 10400, Thailand

^9^Department of Biochemistry, Faculty of Pharmacy, Mahidol University, Bangkok, 10400, Thailand

^10^Biomarker and Epigenetic Science for Targeted Therapeutics (BEST) Research Center, Faculty of Pharmacy, Mahidol University, Bangkok, 10400, Thailand.

#Contributed equally to this work.

***Corresponding authors**

Khanit Sa-ngiamsuntorn, Ph.D.

Department of Biochemistry, Faculty of Pharmacy, Mahidol University, Bangkok, 10400, Thailand.

E-mail: khanit.san@mahidol.ac.th

**Supplementary tables**

**Table S1.** HBV genotypes A-J used in this study were obtained from the NCBI database.

| **GenBank accession no.** | **Organisms** | **Genome length (bp)** | **Definition** | **References** |
| --- | --- | --- | --- | --- |
| HE974362.1 | HBV genotype A1 | 3221 | HBV genotype A1 complete genome, isolate Mart-B01 | (1) |
| HE974364.1 | HBV genotype A2 | 3221 | HBV genotype A2 complete genome, isolate Mart-B15 | (1) |
| AB981583.1 | Hepatitis B virus genotype B | 3215 | HBV genotype B DNA, complete genome, isolate: P2-121214 | (2) |
| LC456132.1 | Hepatitis B virus genotype C | 3215 | Hepatitis B virus genotype C CAM-HB31 DNA, complete genome | (3) |
| HE815465.1 | Hepatitis B virus genotype D | 3182 | HBV genotype D, serotype ayw3, complete genome | Available from: <https://www.ncbi.nlm.nih.gov/nuccore/HE815465.1>  accessed on May 8, 2022 |
| U95551.1 | Hepatitis B virus | 3182 | Hepatitis B virus subtype ayw, complete genome | Available from: <https://www.ncbi.nlm.nih.gov/nuccore/U95551.1>  accessed on May 8, 2022 |
| HE974384.1 | Hepatitis B virus genotype E | 3212 | HBV genotype E complete genome, isolate Mart-B84 | (1) |
| DQ823095.1 | Hepatitis B virus | 3215 | Hepatitis B virus genotype F isolate BA45, complete genome | (4) |
| AB625342.1 | Hepatitis B virus genotype G | 3248 | Hepatitis B virus genotype G DNA, complete genome, isolate: MEX918M | Available from: <https://www.ncbi.nlm.nih.gov/nuccore/383082100>  accessed on May 8, 2022 |
| AB275308.1 | Hepatitis B virus | 3215 | Hepatitis B virus DNA, complete genome, genotype: H | Available from: <https://www.ncbi.nlm.nih.gov/nuccore/AB275308.1>  accessed on May 8, 2022 |
| AF241411.1 | Hepatitis B virus genotype I | 3215 | Hepatitis B virus genotype I isolate 8290, complete genome | (5) |
| AB486012.1 | Hepatitis B virus genotype J | 3182 | Hepatitis B virus genotype J DNA, complete genome, clone: JRB34 | (6) |

**Table S2.** Sequences of primers and probes were used in this study.

| **Primers / probes** | **Sequences (5’-3’)** | **Length (nt)** | **Tm (°C)** | **GC content (%)** |
| --- | --- | --- | --- | --- |
| Cas9_F | acatgtacgtggaccaggag | 20 | 59.10 | 55.0 |
| Cas9_R | tcttcttcaccacctcctcg | 20 | 58.74 | 55.0 |
| HBV DNA_F | GTTGCCCGTTTGTCCTCTAATTC | 20 | 65.3 | 55.0 |
| HBV DNA_R | GGAGGGATACATAGAGGTTCCTTGA | 20 | 68.0 | 55.0 |
| cccDNA_580_ F | GACTCTCTCGTCCCCTTCTC | 20 | 58.6 | 60.0 |
| cccDNA_580_ R | ATGGTGAGGTGAACAATGCT | 20 | 57.4 | 45.0 |
| PRNP_F | GACCAATTTATGCCTACAGC | 20 | 50.0 | 45.0 |
| PRNP_R | TTTATGCCTACAGCCTCCTA | 20 | 50.0 | 45.0 |
| HBV RNA_F | GCACTTCGCTTCACCTCTGC | 20 | 61.9 | 60.0 |
| HBV RNA_R | CTCAAGGTCGGTCGTTGACA | 20 | 60.0 | 55.0 |
| pgRNA_F | TGTTCAAGCCTCCAAGCT | 18 | 48.0 | 50.0 |
| pgRNA_R | GGAAAGAAGTCAGAAGGCAA | 20 | 50.0 | 45.0 |
| preS1 RNA_F | GGGTCACCATATTCTTGGGAAC | 22 | 55.0 | 50.0 |
| preS1 RNA_R | CGAATGCTCCCRCTCCTAC | 18 | 55.0 | 61.0 |
| GAPDH_F | GAAATCCCATCACCATCTTCC | 21 | 64.7 | 47.6 |
| GAPDH_R | AAATGAGCCCCAGCCTTCTC | 20 | 66.9 | 55.0 |
| cccDNA_323_ F (ddPCR) | cttctcatctgccggacc | 18 | 64.0 | 61.1 |
| cccDNA_323_ R (ddPCR) | cacagcttggaggcttga | 18 | 63.4 | 55.6 |
| cccDNA probe (ddPCR) | **FAM**-aggctgtaggcataaattggtct-**BHQ-1** | 23 | 64.0 | 43.5 |
| RPP30_F (ddPCR) | CTGCTTTTGGAATTATCTCTAC | 22 | 52.7 | 36.4 |
| RPP30_R (ddPCR) | GGAAGCTGGAAGACAATC | 18 | 52.7 | 50.0 |
| RPP30_Probe (ddPCR) | **HEX**-AACCTCGGCCATCAGAAGGAGAT-**BHQ-1** | 23 | 63.4 | 52.2 |
| HBV_Fragment_1883_F | TAGCGCCTCATTTTGTGGGT | 20 | 59.9 | 50.0 |
| HBV_Fragment_1883_R | GGAGAAGGGGACGAGAGAGT | 20 | 60.0 | 60.0 |
| HBV_Fragment_717_F | GTATTTCCCTGCTGGTGGCT | 20 | 60.0 | 55.0 |
| HBV_Fragment_717_R | CTTGGCCCCCAATACCACAT | 20 | 60.0 | 55.0 |

**Table S3A.** Confirmation of Off-targets in human genome (Gencode Release 26 (GRCh38.p10) Primary) by SYNTHEGO.

| **gRNAs** | **Guide sequence (5’-3’)** | **On-target gene(s)** | **Strand** | **Off-targets in Human genome** | **Chromosome** |
| --- | --- | --- | --- | --- | --- |
| gRNA1 | Taccgcagagtctagactcg | Not found | | | |
| gRNA2 | CACCACGAGTCTAGACTCTG | Not found | | | |
| gRNA3 | accccttctcgtgttacagg | Not found | | | |
| RT gRNA | tttcagttatatggatgatg | Not found | | | |
| ANKRD gRNA | TCTGACAAGGGCTGTAAGTG | FANCI | + | 0-0-0-13-246 | 1, 5, 6, 8, 9, 12, 19, 20, X |

**Table S3B.** Confirmation of Off-targets in human genome (GRCh38) by Find off-targets by seq (sanger.ac.uk).

| **gRNAs** | **Guide sequence (5’-3’)** | **PAM sequence** | **Off-targets in Human genome** |
| --- | --- | --- | --- |
| gRNA1 | Taccgcagagtctagactcg | NGG | 0-0-0-0-26 |
|  |  | CCN | 0-0-0-0-30 |
| gRNA2 | CACCACGAGTCTAGACTCTG | NGG | 0-0-0-3-87 |
|  |  | CCN | 0-0-1-5-84 |
| gRNA3 | accccttctcgtgttacagg | NGG | 0-0-0-2-43 |
|  |  | CCN | 0-0-0-4-75 |
| RT gRNA | tttcagttatatggatgatg | NGG | 0-0-1-13-209 |
|  |  | CCN | 0-0-0-46-941 |
| ANKRD gRNA | TCTGACAAGGGCTGTAAGTG | NGG | 1-0-0-13-246 |
|  |  | CCN | 0-0-0-7-105 |

**Supplementary figures**


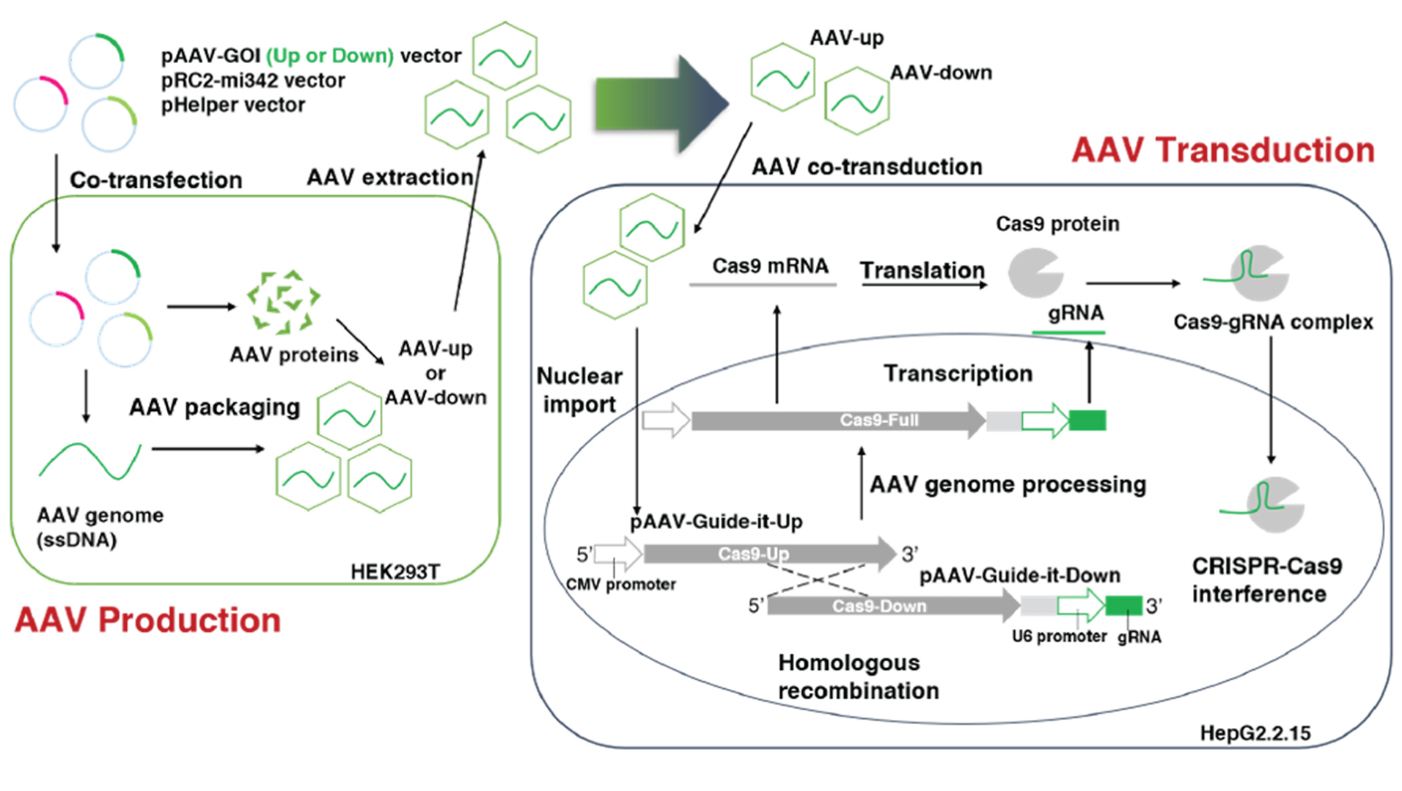


**Fig. S1. AAV-mediated delivery of the CRISPR/Cas9 system into hepatocytes.** (A) Diagram illustrating AAV vector production in HEK293T cells and CRISPR/Cas9 expression in transduced HepG2.2.15 cells. Homologous recombination generated a full-length Cas9 gene driven by the CMV promoter. Both Cas9 and gRNA sequences were transcribed, followed by translation of Cas9 mRNA into the Cas9 endonuclease. Cas9 formed a complex with gRNAs to mediate targeted genome editing in HepG2.2.15 cells.


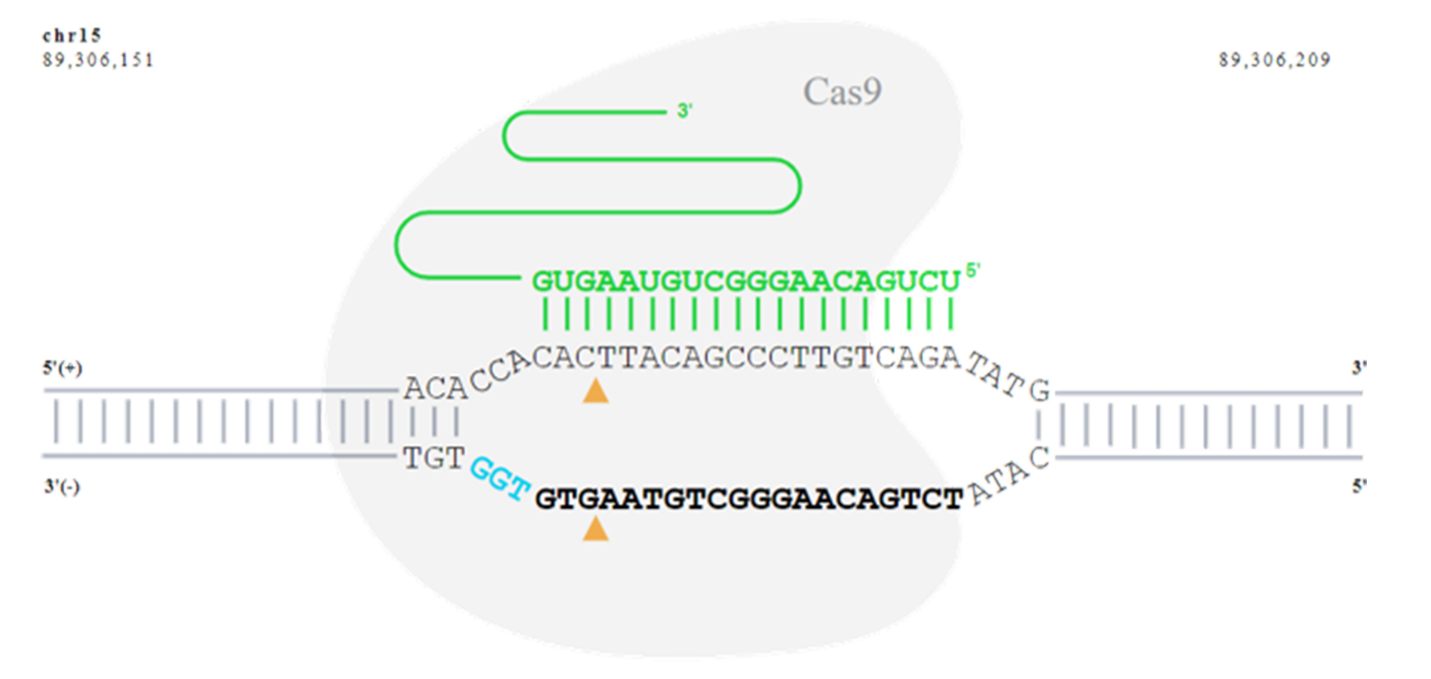


**Fig. S2.** **Genomic location of the on-target site for the ANKRD gRNA in the human genome, predicted by SYNTHEGO.** The gRNA sequence, PAM site, and target DNA are shown in green, blue, and bold font, respectively. Orange arrows indicate the predicted double-stranded break (DSB) site. The chromosome containing the target site is shown in the top-left corner. Numbers at the top-left and top-right denote the genomic coordinates of the displayed region.


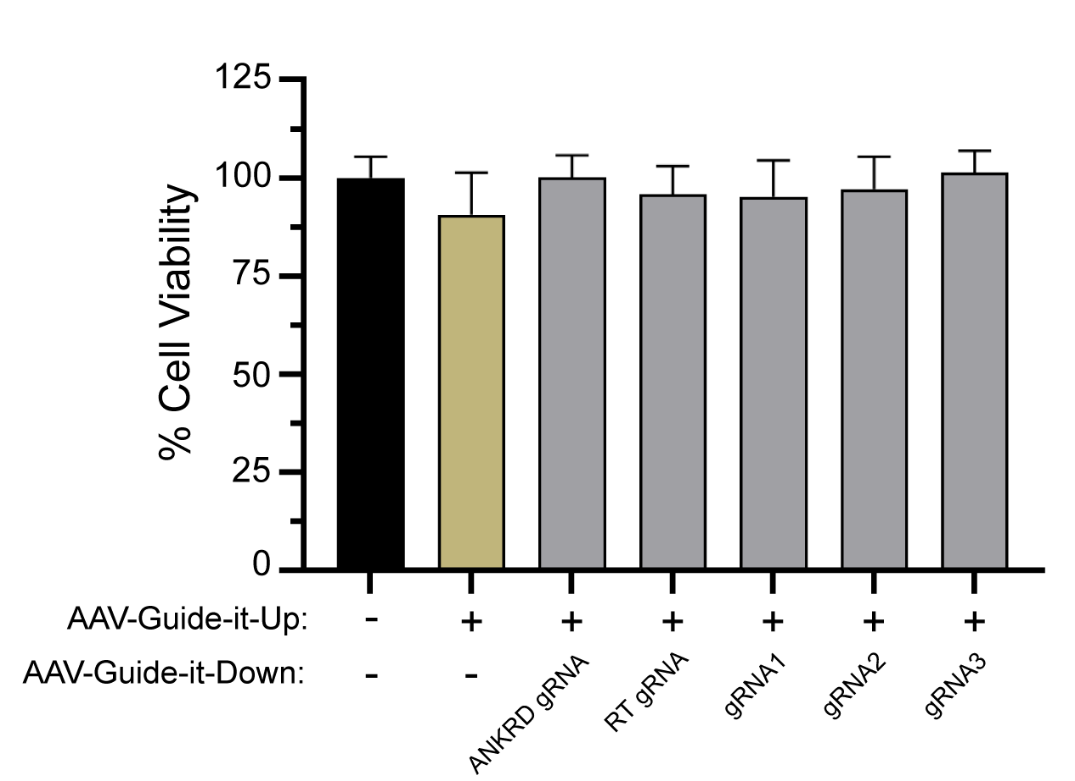


**Fig. S3. Evaluation of cytotoxicity in imHC cells following treatment with AAV-delivered CRISPR/Cas9 carrying the designed guide RNAs using the MTT assay.** imHC cells (3 × 10⁴ cells/well) were seeded in 96-well plates and incubated overnight at 37 °C in a humidified atmosphere containing 5% CO₂. Cells were subsequently transduced with the dual AAV system (AAV-up and AAV-down) for 24 h. Following transduction, the culture medium was replaced with fresh complete medium, and cells were maintained for an additional 6 days prior to cytotoxicity assessment. Cell viability was determined using the MTT assay. Cell viability (%) was expressed as mean ± SD from eight replicates for each condition and normalized to the untreated control group. No significant cytotoxic effects were observed in cells treated with the AAV-delivered CRISPR/Cas9 system under the experimental conditions used in this study.


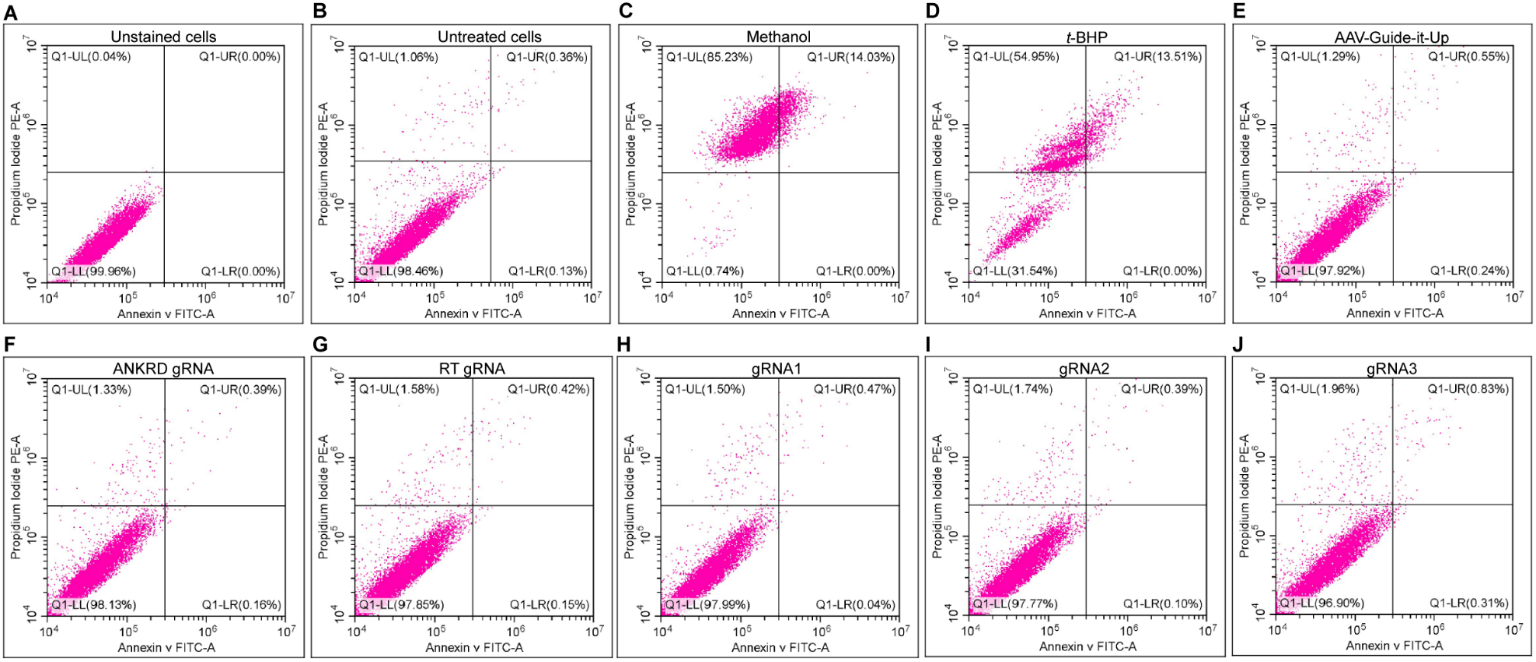


**Fig. S4. Evaluation of apoptosis-associated cytotoxicity in imHC cells following AAV-delivered CRISPR/Cas9 treatment by Annexin V/PI staining.** imHC cells (1 × 10⁶ cells/well) were seeded in 6-well plates and incubated overnight at 37 °C in a humidified atmosphere containing 5% CO₂. Cells were subsequently transduced with the dual AAV system (AAV-up and AAV-down) for 24 h. After transduction, the medium was replaced with fresh complete medium, and cells were maintained for an additional 6 days prior to apoptosis analysis. Apoptotic cell death was assessed using Annexin V/PI staining. Experimental controls included unstained cells (A), Annexin V/PI–stained untreated cells (B), cold methanol-fixed cells (C), and tert-butyl hydroperoxide (t-BHP)-treated cells as a positive control for apoptosis induction (D). Additional treatment groups included cells transduced with AAV-up alone (E) and cells transduced with the dual AAV system carrying ANKRD gRNA (F), RT gRNA (G), gRNA1 (H), gRNA2 (I), or gRNA3 (J). No significant apoptosis-associated cytotoxicity was observed in CRISPR/Cas9-treated cells under the experimental conditions used in this study.


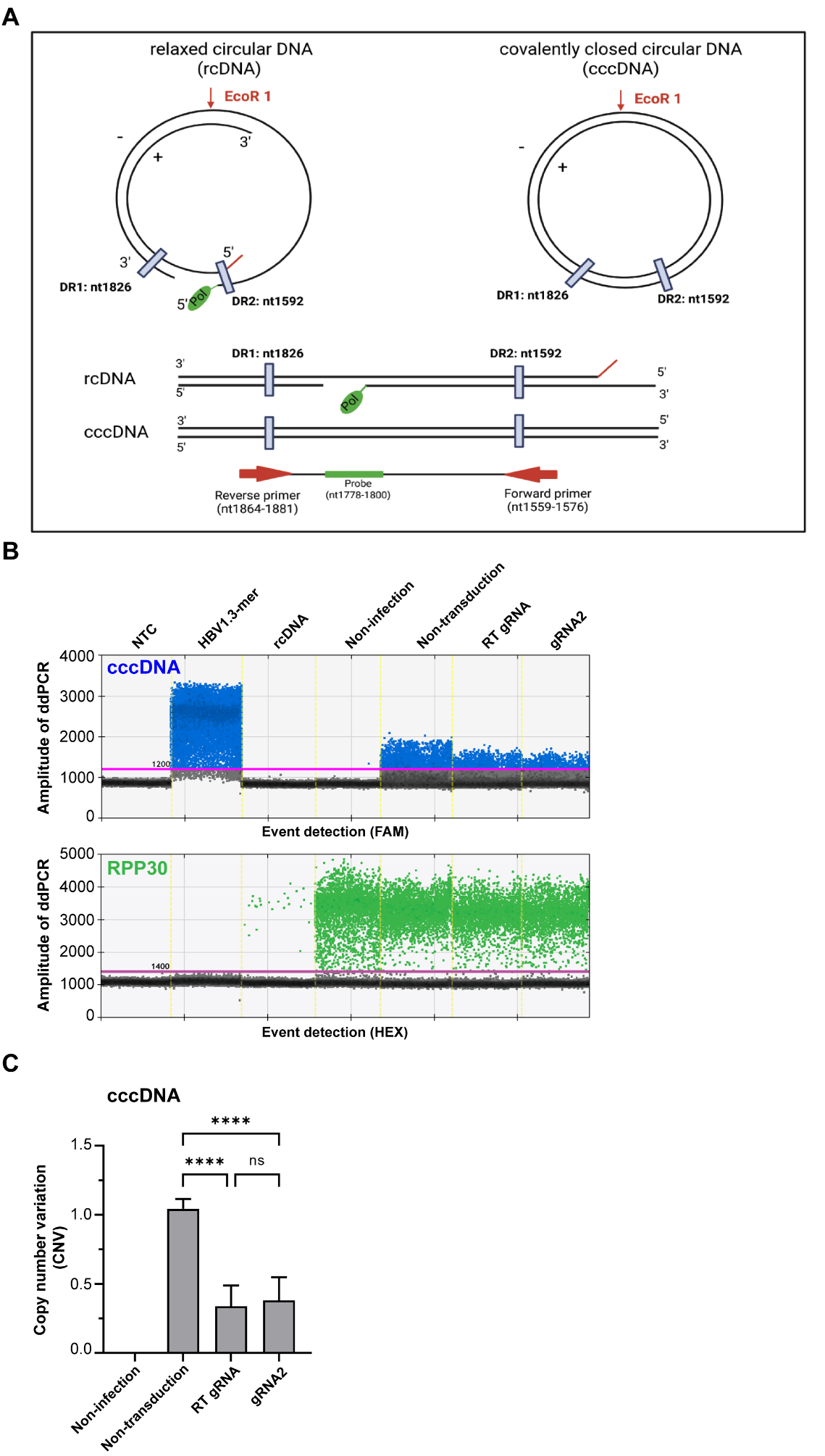


**Fig. S5. Quantification of HBV cccDNA-associated signals using probe-based droplet digital PCR (ddPCR).** Probe-based ddPCR was employed to quantify HBV cccDNA-associated signals in HBV-infected cells following treatment with AAV-delivered CRISPR/Cas9 carrying the designed guide RNAs. Detection and quantification of HBV cccDNA by ddPCR were performed as previously described (7). A schematic representation of the HBV genome in the forms of relaxed circular DNA (rcDNA) and covalently closed circular DNA (cccDNA), adapted from a previous report (7), is shown in (A). Two direct repeat regions (DRs) are located at nucleotides (nt) 1826 and 1592, with numbering initiated from the EcoRI site. The rcDNA genome contains a complete minus strand with a nine-base terminal redundancy and a covalently attached viral polymerase at the 5′ end (green oval). In contrast, the plus strand contains a defined 5′ end with an RNA primer (red line) and a variable 3′ end. Primers for cccDNA amplification were selectively designed to flank the single-stranded gap region present in rcDNA. Combined with a cccDNA-selective probe targeting this region, the assay enabled preferential detection and quantification of cccDNA-associated signals by ddPCR. Representative droplet scatter plots demonstrating quantification of cccDNA and the reference gene (RPP30) in the HBV-infected imHC model are shown in (B). Multiple controls were included, including a non-template control, HBV 1.3-mer plasmid as a positive control, HBV DNA extracted from HBVcc representing the rcDNA form, and a non-infected control. Experimental groups included HBV-infected imHC cells with or without AAV-mediated transduction carrying RT gRNA or gRNA2. HBV cccDNA-associated signals were quantified as copy number variation (CNV) normalized to the reference gene RPP30 (C). Data are presented as mean ± SD. *, **, ***, and **** indicate statistical significance at *p* < 0.05, *p* < 0.01, *p* < 0.001, and *p* < 0.0001, respectively.

**References**

1. Brichler S, Lagathu G, Chekaraou MA, Le Gal F, Edouard A, Deny P, et al. African, Amerindian and European hepatitis B virus strains circulate on the Caribbean Island of Martinique. Journal of General Virology. 2013;94(10):2318-29.

2. Kamitsukasa H, Iri M, Tanaka A, Nagashima S, Takahashi M, Nishizawa T, et al. Spontaneous reactivation of hepatitis B virus (HBV) infection in patients with resolved or occult HBV infection. Journal of medical virology. 2015;87(4):589-600.

3. Chuon C, Takahashi K, Matsuo J, Katayama K, Yamamoto C, Ko K, et al. High possibility of hepatocarcinogenesis in HBV genotype C1 infected Cambodians is indicated by 340 HBV C1 full-genomes analysis from GenBank. Scientific reports. 2019;9(1):12186.

4. Torres C, y Leone FGP, Pezzano SC, Mbayed VA, Campos RH. New perspectives on the evolutionary history of hepatitis B virus genotype F. Molecular phylogenetics and evolution. 2011;59(1):114-22.

5. Hannoun C, Norder H, Lindh M. An aberrant genotype revealed in recombinant hepatitis B virus strains from Vietnam. Journal of General Virology. 2000;81(9):2267-72.

6. Tatematsu K, Tanaka Y, Kurbanov F, Sugauchi F, Mano S, Maeshiro T, et al. A genetic variant of hepatitis B virus divergent from known human and ape genotypes isolated from a Japanese patient and provisionally assigned to new genotype J. Journal of virology. 2009;83(20):10538-47.

7. Thongsri P, Pewkliang Y, Borwornpinyo S, Wongkajornsilp A, Ruenraroengsak P, Anurathapan U, et al. Panduratin A from Boesenbergia rotunda suppresses hepatitis B virus by targeting HNF1alpha and synergizing with antiviral agents. Chin Med. 2026;21(1):10.
